# Protein-lysine methyltransferases G9a and GLP1 promote responses to DNA damage

**DOI:** 10.1038/s41598-017-16480-5

**Published:** 2017-11-30

**Authors:** Vasudeva Ginjala, Lizahira Rodriguez-Colon, Bratati Ganguly, Prawallika Gangidi, Paul Gallina, Husam Al-Hraishawi, Atul Kulkarni, Jeremy Tang, Jinesh Gheeya, Srilatha Simhadri, Ming Yao, Bing Xia, Shridar Ganesan

**Affiliations:** 1 Department of Medicine, Rutgers Cancer Institute of New Jersey, Rutgers University, 195 Little Albany street, New Brunswick, New Jersey, 08903 USA; 2 Cornell University, College of Engineering, Department of Biological Engineering, 111 Wing Drive, Ithaca, NY, 14853-5701 USA

## Abstract

Upon induction of DNA breaks, ATM activation leads to a cascade of local chromatin modifications that promote efficient recruitment of DNA repair proteins. Errors in this DNA repair pathway lead to genomic instability and cancer predisposition. Here, we show that the protein lysine methyltransferase G9a (also known as EHMT2) and GLP1 (also known as EHMT1) are critical components of the DNA repair pathway. G9a and GLP1 rapidly localizes to DNA breaks, with GLP1 localization being dependent on G9a. ATM phosphorylation of G9a on serine 569 is required for its recruitment to DNA breaks. G9a catalytic activity is required for the early recruitment of DNA repair factors including 53BP and BRCA1 to DNA breaks. Inhibition of G9a catalytic activity disrupts DNA repair pathways and increases sensitivity to ionizing radiation. Thus, G9a is a potential therapeutic target in the DNA repair pathway.

## Introduction

Many important biological processes are regulated by post-translational modifications. Some of these, histone modification by phosphorylation, acetylation, or ubiquitylation have been linked to the repair of damaged DNA^1–4^. Dynamic changes in histone methylation has also been implicated as part of the DNA repair response and several histone methyltransferases and demethylases, have been identified as regulating this process^5,6^.

Specific lysine methylation of N-terminus histone tails can serve as either a mark of transcriptional active euchromatin or silent heterochromatin. Histone H3 methylation of H3 lysine 4, H3 lysine 36, and H3 lysine 79 has been associated with transcriptional activation whereas, methylation of Histone H3 lysine 9, H3 lysine 27, and H4 lysine 20 are usually linked with transcriptional repression. G9a (also known as EHMT2), and the closely related GLP1 (also known as EHMT1) are ubiquitously expressed protein methyl transferases that contain a Su(var), Enhancer of Zeste, Trithorax (SET) domain^7,8^, and localizes in euchromatin regions. Both, G9a and GLP1 primarily catalyze the mono- and di-methylation of histone H3 lysine 9 (H3K9me1/H3K9me2), although they also can methylate histone H1 and H3 lysine 27^9–11^ and histone H3 lysine 56 (H3K56)^12^. They also have several other non-histone protein substrates including p53^4,13^. G9a has been reported to be dysregulated in different types of cancer and its overexpression has been associated with poor prognosis^14–16^. Loss of either G9a or GLP1 in the mouse leads to embryonic lethality^17,18^, demonstrating they play critical roles in development.

Both G9a and GLP1 are phosphorylated by ATM kinase, and catalytic inhibition of G9a leads to genomic instability^19^, suggesting they play a role in the DNA damage response (DDR)^20^. However, the direct role of G9a and GLP1 in DNA repair is far from clear. In this study, we show that phosphorylation of G9a on serine 569 by ATM leads to its recruitment to sites of DNA breaks. We further demonstrate that G9a catalytic activity is required for the early H2AX-independent recruitment of 53BP1 and BRCA1 but dispensable for late recruitment of these proteins. Loss of G9a or its catalytic inhibition impairs both HR and NHEJ and leads to radio-sensitivity. These findings establish G9a as a potentially pharmacologically targetable component of the DNA repair pathway.

## Results

### G9a and GLP1 are recruited to DNA-damage sites

**Figure 1:**
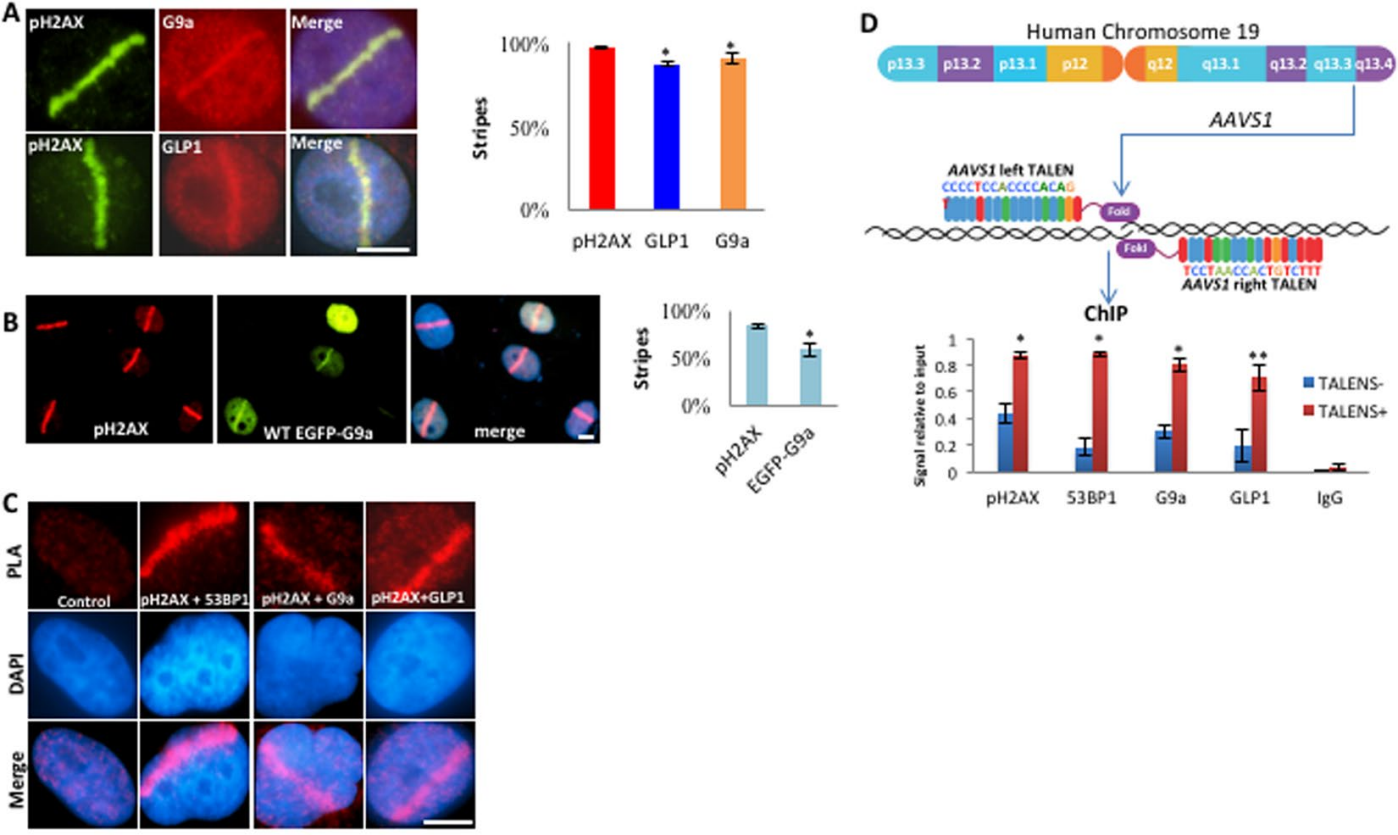
G9a and GLP1 accumulate at DNA-damage sites. (**A**) HeLa cells were laser micro-irradiated and after 10 minutes processed for IF staining using indicated antibodies. (**B**) HeLa cells co-transfected with GFP-G9a were micro-irradiated IF staining for H2AX and GFP signal are shown. (**C**) PLA was used to visualize regions of close proximity between γH2AX and either 53BP1, G9a or GLP1 in U2OS cells treated with micro-irradiation. PLA using only γH2AX antibody alone is shown as negative control. (**D**) U2OS cells were transfected with either TALEN targeting the AAVS1 site and having intact FOK1-nuclease (TALEN + ) or vectors lacking active FOK1-nuclease domains (TALEN−) and processed for ChIP using antibodies indicated. PCR was conducted using primers in the regions flanking AAVS1 site, and signals were quantified and plotted for each condition. For panels A and B quantification of IF images are shown, plotted as % of micro-irradiated cells that were also positive for pH2AX, G9a, GLP1, and EGFP-G9a. Data represent 100micro-irradiated cells in three independent experiments and data are expressed as the mean ± SEM. **P < 0.01, *P < 0.05. Scale bar, 5 μm.

To investigate localization of G9a and GLP, UV-laser scissors were used to create specific sub-nuclear region of DNA breaks^21^, and G9a and GLP were localized by immunofluorescence using antibodies recognizing the endogenous proteins. We found that the endogenous G9a and GLP1 rapidly localized to sites of DNA damage induced by laser scissors in U2OS cells, being detectable within 2 minutes and remaining present up to 24 hours after induction of breaks (Fig. 1A, Supplemental Figs 1 and 2). To confirm this finding, U2OS cells were transfected with GFP-tagged human G9a. Exogenous GFP-tagged G9a also showed rapid recruitment to DNA breaks (Fig. 1B). The close co-localization of G9A and GLP1 with γ-H2AX was then confirmed using a proximity-ligation assay^22^ (Fig. 1C).

To directly interrogate localization of G9a and GLP to a DNA double-strand break induced at a specific genomic location, engineered transcription activator-like effector nuclease (TALEN)^23^ targeting the AAVS1^24^ site on chromosome 19 were employed. As expected chromatin-immunoprecipitation (ChIP) demonstrated that induction of DNA breaks by expression of TALEN, led to enrichment of both γ-H2AX and 53BP1 in region flanking the AAVS1 target site (Fig. 1D). ChIP using antibodies to endogenous proteins also demonstrated that both G9a and GLP1 had increased occupancy in chromatin regions flanking the AAVS1 site upon induction of DNA breaks. These findings demonstrate that G9a and GLP are recruited to sites of engineered DNA double strand breaks.

### G9a is required for GLP localization to DNA breaks

**Figure 2:**
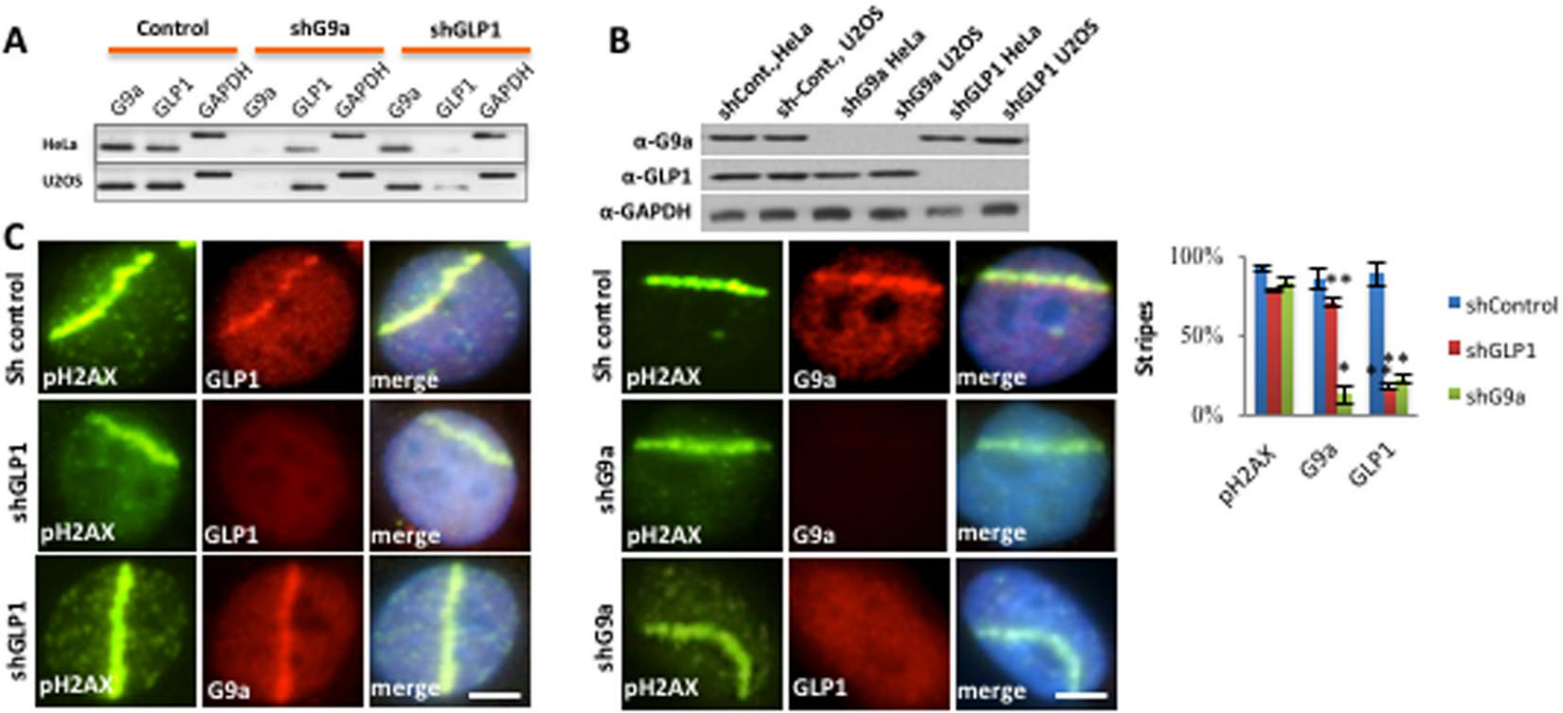
G9a is required for GLP localization to DNA breaks. (**A**) Total RNA was extracted from Hela and U2OS cells expressing control, G9a or GLP1 shRNAs and subjected to RT-PCR using primers for G9a, GLP1, and GAPDH. (**B**) Western blots of HeLa and U2OS cells treated as above, probed with indicated antibodies. (**C**) U2OS cells expressing control shRNA, GLP1 shRNA (Left panels) or G9a shRNA (right panels) were laser micro-irradiated and analyzed by IF as indicated. Quantifications of results are plotted on right. Each point is the mean of at least three experiments ± the SEM. **P < 0.01, *P < 0.05. Scale bar, 5 μm.

G9a is reported to interact directly with GLP1^18^. To dissect the functional relationship between G9a and GLP1, shRNAs specific for either G9a or GLP1 were utilized (Fig. 2A,B). The specificity of the G9a and GLP1 shRNA affecting RNA and protein levels of the target gene was confirmed using RT-PCR (Fig. 2A) and western blotting (Fig. 2B). HeLa cells treated with control, G9a or GLP1 specific shRNA were then treated with laser scissors and localization of endogenous G9a and GLP visualized by immunofluorescence. Knockdown of G9a led to loss of GLP1 localization at DNA breaks, while knockdown of GLP1 did not affect G9a localization (Fig. 2C). Knockdown of G9a can affect protein levels of GLP1, so the effect of G9a loss on GLP1 localization may be in part due to decreased GLP1 expression. However, our data suggests that GLP1 recruitment to DNA breaks is in part dependent upon G9a, but that G9a is recruited independently of GLP1.

### G9a localization is dependent upon ATM activation

**Figure 3:**
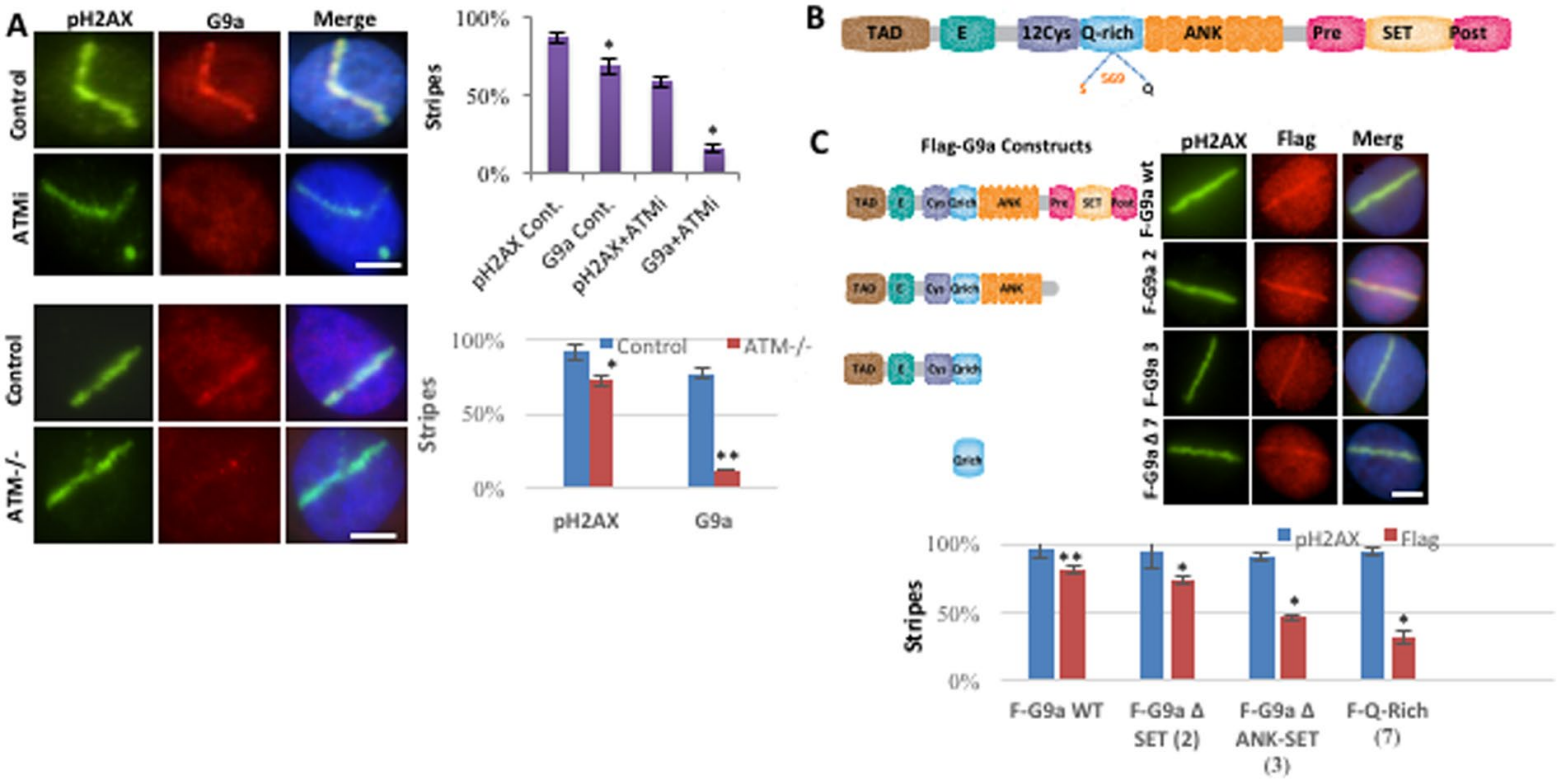
G9a localization is dependent upon ATM activation. (**A**) U2OS cells treated with ATMi, 0.5 uM overnight, or vehicle (Top panels) and ATM −/− and ATM + / + human fibroblast cells (bottom panels) cells were subjected to micro-irradiation and processed for IF using indicated antibodies. (**B**) Schematic representation of the domains of G9a protein. (**C**) U2OS cells stably expressing Flag-G9a deletion constructs were treated with micro-irradiation, and localization at DNA breaks are shown and quantified as indicated. Data are expressed as the mean ± SEM of at least three experiments. **P < 0.01, *P < 0.05. Scale bar, 5 μm.

G9a contains an SQ site at serine 569 that has been identified as an ATM/ATR phosphorylation site^20^. To determine the role of ATM in recruitment of G9a to DNA damage site, both pharmacologic and genetic approaches were used. Treatment with an ATM inhibitor (KU55933), but not an ATR inhibitor (VE-821)^25^ reduced G9a and GLP1 recruitment to sites of laser scissors induced DNA breaks (Fig. 3A, Supplemental Fig. 3, panels A,B). Similarly, G9a recruitment was absent in ATM −/− fibroblasts when compared to wt cells (Fig. 3A). Interestingly G9a and GLP1 recruitment to DNA breaks was intact in H2AX−/− mouse embryonic fibroblasts (MEFs) and MDC1−/− MEFs (Supplemental Figs 4 and 5), suggesting that G9a/GLP recruitment is dependent on ATM activity, but not on presence of H2AX or MDC1.

### Phosphorylation of G9a on S569 is required for G9a recruitment to DNA breaks

G9a has an SQ rich region, an Ankyrin repeat domain thought to mediate binding to methylated histone^26,27^, and the catalytic SET domain (Fig. 3B). To investigate what regions of G9a are required for localization to DNA breaks, a series of FLAG-tagged deletion constructs were generated. These constructs were introduced into HeLa cells, and localization to DNA breaks was evaluated using immunofluorescence using FLAG antibody. Deletion constructs that lacked the SET domain and the Ankyrin repeat domains still localized to induced DNA breaks (Fig. 3C), although with reduced efficiency. This suggests that although the SET domains and Ankyrin repeats may contribute to efficient localization, they are not absolutely required. The small Q-rich region of G9a alone did show some recruitment to DNA breaks (Fig. 3C). The region contains an SQ site at serine 569, previously identified as an ATM/ATR phosphorylation site^20^.

**Figure 4:**
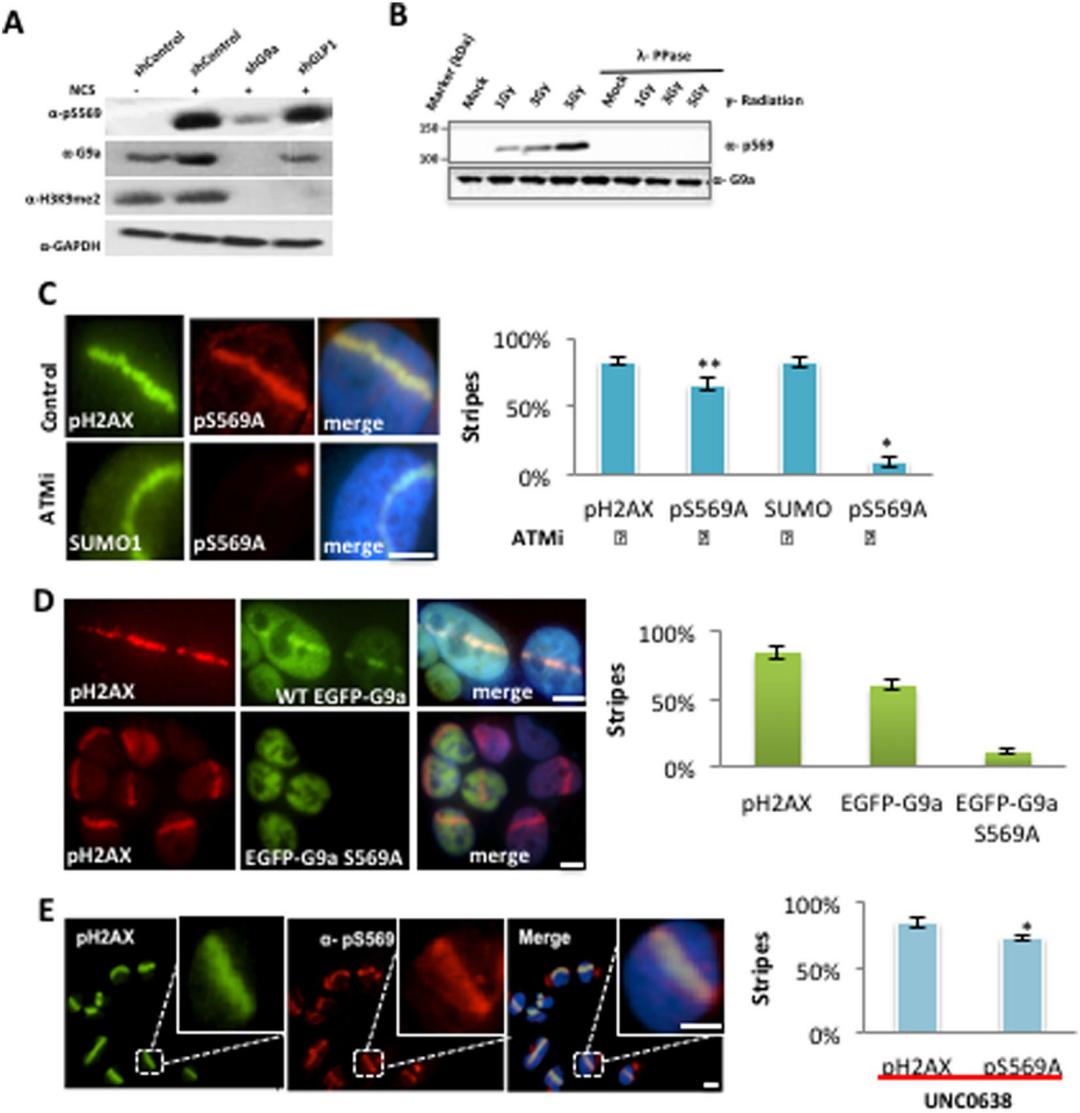
Phosphorylation of G9a on S569 is required for G9a recruitment to DNA breaks. (**A**) Western blots of U2OS cells expressing control or indicated shRNA, and NCS or vehicle, probed with indicated antibodies. (**B**) Western blots of U2OS cells treated with IR and probed with antibodies against pS569-G9a or total G9a. L-phosphatase treatment of extracts is as labeled. (**C**) U2OS cells treated with control or ATMi, treated with micro-irradiation and processed for IF using indicated antibodies. (**D**) U2OS cells were transfected with either EGFP-G9a or EGFP-G9a S569A mutant, and treated with micro-irradiation. Localization of endogenous pH2AX (red) and ectopic GFP-fusion proteins (green) are shown. E, U2OS cells were treated with UNC0638, and localization of pH2AX and pG9A S569 to regions of micro-irradiation are shown. Data are expressed as the mean ± SEM of at least three experiments. **P < 0.01, *P < 0.05. Scale bar, 5 μm.

To investigate the role of phosphorylation of G9a at S569, affinity-purified phospho-specific antibodies to this residue were generated. This antibody recognized a band consistent with phosphorylated G9a in cells treated with either γ-radiation or Neocarzinostatin (NCS), but not in untreated cells (Fig. 4A,B). Knockdown of G9a but not GLP1 abolished this band; efficacy of G9a and GLP knock down were confirmed by their effect on global H3K9me2 levels. λ-phosphatase treatment also abolished this band, confirming the antibody is specific for phosphorylated G9a (Fig. 4B). Furthermore G9a phospho-specific antibodies recognize wt-G9a-GFP after NCS treatment, but not phospho-mutant S569A G9a-GFP (Supplemental Fig. 3C), demonstrating this antibody is specific for the S569 phosphorylation. Immunofluorescence experiments using this phospho-G9a S569 Ab demonstrate that pS569-G9a is rapidly recruited to sites of DNA breaks (Fig. 4C) and the signal was abolished by treatment with an ATM inhibitor. Treatment with the G9a catalytic inhibitor UNC0638^28^ affected neither G9a phosphorylation nor its localization to DNA breaks (Fig. 4E), demonstrating that catalytic activity of G9a is not required for its phosphorylation or its recruitment to DNA breaks.

To evaluate the role of G9a-S569 phosphorylation in recruitment of G9a to DNA breaks, a GFP-tagged G9a S569A phospho-mutant was expressed in HeLa cells. Unlike the wild type (wt) G9a-GFP, the pS569A G9a-GFP mutant failed to recruit to sites of DNA breaks induced by laser scissors (Fig. 4D). These data demonstrate that G9a is phosphorylated on S569 by ATM upon induction of G9a, and that this phosphorylation is required for its recruitment to DNA breaks.

### G9a is required for early recruitment of 53BP1 to DNA breaks

**Figure 5:**
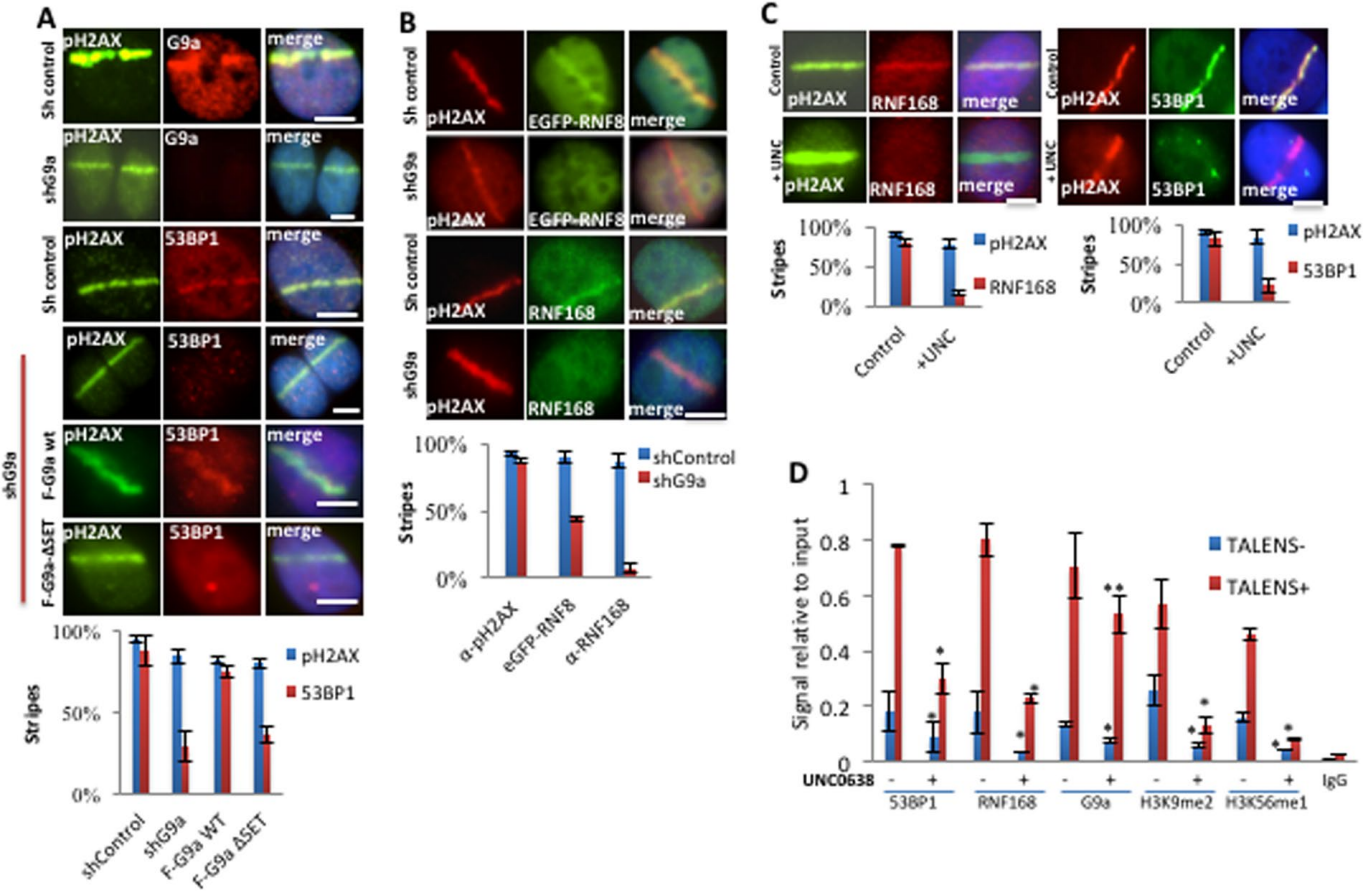
G9a is required for early recruitment of 53BP1 to DNA breaks. (**A**,**B**) U2OS cells expressing control or G9a shRNA, and expressing Flag or EGFP tagged vectors were laser micro-irradiated, and after ~5–10 minutes and analyzed by IF as indicated. Quantifications of results are shown under the images. (**C**) U2OS cells were mock or UNC0638 treated, micro-irradiated and processed for IF using indicated antibodies. Quantified results are shown below. (**D**) U2OS cells were transfected with either TALEN vectors targeting the AAVS1 site (TALEN + ), or TALEN vectors lacking active FOK1 nuclease domain (TALEN−) and processed for ChIP using antibodies as shown. Each point is the mean of at least three experiments ± the SEM. **P < 0.01, *P < 0.05. Scale bar, 5 μm.

To identify the role of G9a in the DNA repair signaling pathway, the effect of G9a knockdown on recruitment of DNA repair factors was investigated. G9a knockdown abrogated recruitment of 53BP1 normally seen at 10 min after induction of DNA breaks by UV-laser scissors (Fig. 5A). This effect was rescued by an shRNA-resistant cDNA expressing wt G9a but not a mutant G9a lacking the SET domain, suggesting that the catalytic activity of G9a is required for immediate 53BP1 recruitment after DNA damage (Fig. 5A). Consistent with this finding, catalytic inhibition of G9a activity with UNC0638 also led to abolishment of 53BP1 recruitment at this time point (Fig. 5C). 53BP1 recruitment is known to be dependent on RNF168-mediated histone ubiquitination^29^. G9a knockdown led to reduced accumulation of both RNF8 and RNF168 at DNA breaks at 10 min (Fig. 5b). Inhibition of G9a catalytic activity with UNC0638 produced a similar effect on RNF168 (Fig. 5C), suggesting that G9a catalytic activity is required for recruitment of RNF168. Consistent with loss of RNF168 localization, catalytic inhibition of G9a activity also led to loss of K63-linked-polyubiquitination signal normally induced at DNA breaks (Supplemental Fig. 6B). Further, we assayed if another G9a interactor, SPOC1, which also localizes to DNA breaks^30^, is dependent on G9a catalytic activity. G9a catalytic inhibition abrogates SPOC1 localization to DNA breaks (Supplemental Fig. 6C), suggesting that SPOC1 localization to DNA breaks is dependent on G9a activity.

To complement the laser scissors experiments, the effect of G9a catalytic inhibition on the accumulation of DNA repair factors at a TALEN-induced DNA break at the AAVS1 locus was investigated. Treatment with UNC0638 significantly reduced accumulation of 53BP1 and RNF168 at TALEN-induced DNA break as assayed by ChIP (Fig. 5D). H3K9-me2, a known substrate of G9a^18^, was induced was also induced at DNA breaks. Some enrichment of H3K56-me1, another substrate of G9a is also seen^12^, suggesting this modification may also be induced at breaks. Treatment with UNC0638 reduced both the baseline level of H3K9-me2 and H3K56-me1 reduced their induction by DNA breaks when compared with control treated cells. However, consistent with other results, G9a occupancy at DNA breaks is not impaired by UNC0638 treatment (Fig. 5D).

### G9a is not required for late recruitment of repair factors

**Figure 6:**
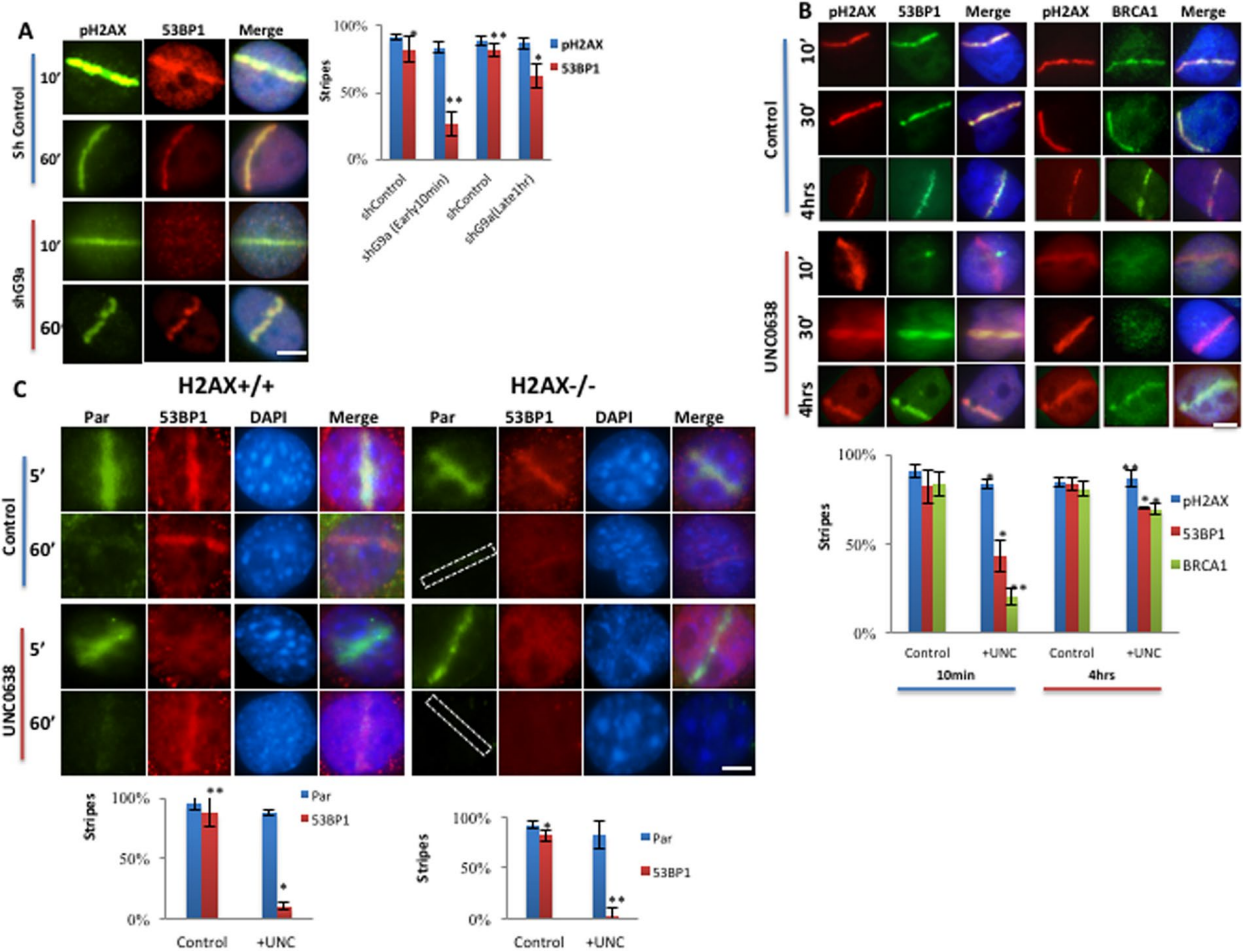
G9a is not required for late recruitment of 53BP1. (**A**) U2OS cells expressing control or shG9a were laser micro-irradiated and after indicated time points, analyzed by IF. Quantifications of results are shown on right. (**B**) U2OS cells were mock or UNC0638 treated, micro-irradiated and at indicted timepoints processed for IF using antibodies shown. Quantifications are shown at bottom. (**C**) H2AX−/− or wt MEFs were mock or UNC0638 treated, micro-irradiated, harvested at different time points, processed for IF and probed as indicated. Quantifications for 5 minute time-point are shown bottom of the images. Each point is the mean of at least three experiments ± the SEM. **P < 0.01, *P < 0.05. Scale bar, 5 μm.

The experiments described so far all examined early time-points after induction of DNA breaks. However early and late recruitment of DNA repair factors may be regulated by different pathways. To determine the effect of G9a inhibition on the dynamics of DNA repair factors recruitment, a detailed time course analysis was performed. In HeLa cells, 53BP1 is normally rapidly recruited to laser-induced DNA damage within 5 min of induction and persists for hours (Supplemental Fig. 7). Either knockdown or catalytic inhibition of G9a led to loss of 53BP1 recruitment seen at 10 minutes, but not later time points (30 min, 4 hrs) (Fig. 6A,B, additional time points presented in Supplemental Fig. 6B).

To determine the effect of G9a inhibition on the dynamics of BRCA1 recruitment to DNA breaks, similar experiments were performed. Endogenous BRCA1 is recruited more slowly than 53BP1 to sites of induced breaks, although appreciable at 5 min (Supplemental Fig. 6B), it is more robust at 10 min after induction of breaks (Fig. 6B,). Catalytic inhibition of G9a also led to loss of recruitment of BRCA1 seen, at 10 min, and 30 minutes, while late recruitment of BRCA1 at 4 hrs. was unaffected (Fig. 6, panel B and, additional time points shown in Supplemental Fig. 6A).

We next analyzed the effect of G9a inhibition on IRIF formation. 53BP1 is recruited to IRIF rapidly in U2OS cells, being seen 30 minutes after IR (Supplemental Fig. 7) and persisting for hours, while BRCA1 has slower IRIF formation, with robust foci being seen only 30–60 minutes after irradiation and persisting for hours. Treatment with UNC0638 led to reduction of foci formation of 53BP1 at 30 minutes, when compared with untreated cells, but no affect at later time points, with recovery seen at 1hr and 4 hours (Supplemental Fig. 7). Treatment with UNC0638 led to decreased foci formation by BRCA1 at 1 hour but foci formation at 4 hours was intact (Supplemental Fig. 7). Thus, Inhibition of G9a activity leads to loss of early recruitment of 53BP1 and BRCA1 to both UV-laser induced breaks and IRIF, but 53BP1 has faster dynamics of loss and recovery than BRCA1. BARD1 also behaves similarly to BRCA1 (Supplemental Fig. 8).

These data demonstrate that G9a activity is required for the early recruitment of 53BP1to DNA breaks but dispensable for the late recruitment. Interesting it has been shown that H2AX is dispensable for early recruitment of 53BP1 to DNA breaks, but is required for late recruitment^31^ (Fig. 6C). To determine the contribution of G9a activity to the early, H2AX-indpendent recruitment of 53BP1 to DNA breaks, experiments were performed in H2AX −/− and H2AX + / + MEFs. As previously shown, H2AX −/− cells had intact early recruitment (5 min) of 53BP1 to laser scissors induced DNA breaks, but failed to support 53BP1 accumulation at later time points (4 hours), when compared with H2AX + / + MEFS. Treatment with UNC0638 abolished this early recruitment of 53BP1 in both H2AX + / + and H2AX−/− MEFs (Fig. 6C). Similarly, 53BP1 IRIF formation was intact in H2AX−/− cells at early time points, but not present at later time points, when compared to H2AX + / + cells^32^ (Supplemental Fig. 9). Treatment with UNC0638 abolished early recruitment of 53BP1 to IRIF in both H2AX−/− and H2AX + / + MEFS, however the late recruitment in UNC0638 treated cells was intact only in H2AX + / + MEFS. This observation suggests G9a and H2AX play complementary roles in early and late recruitment of 53BP1 to DNA breaks.

### G9a promotes HR and non-homologous end-joining (NHEJ) repair

**Figure 7:**
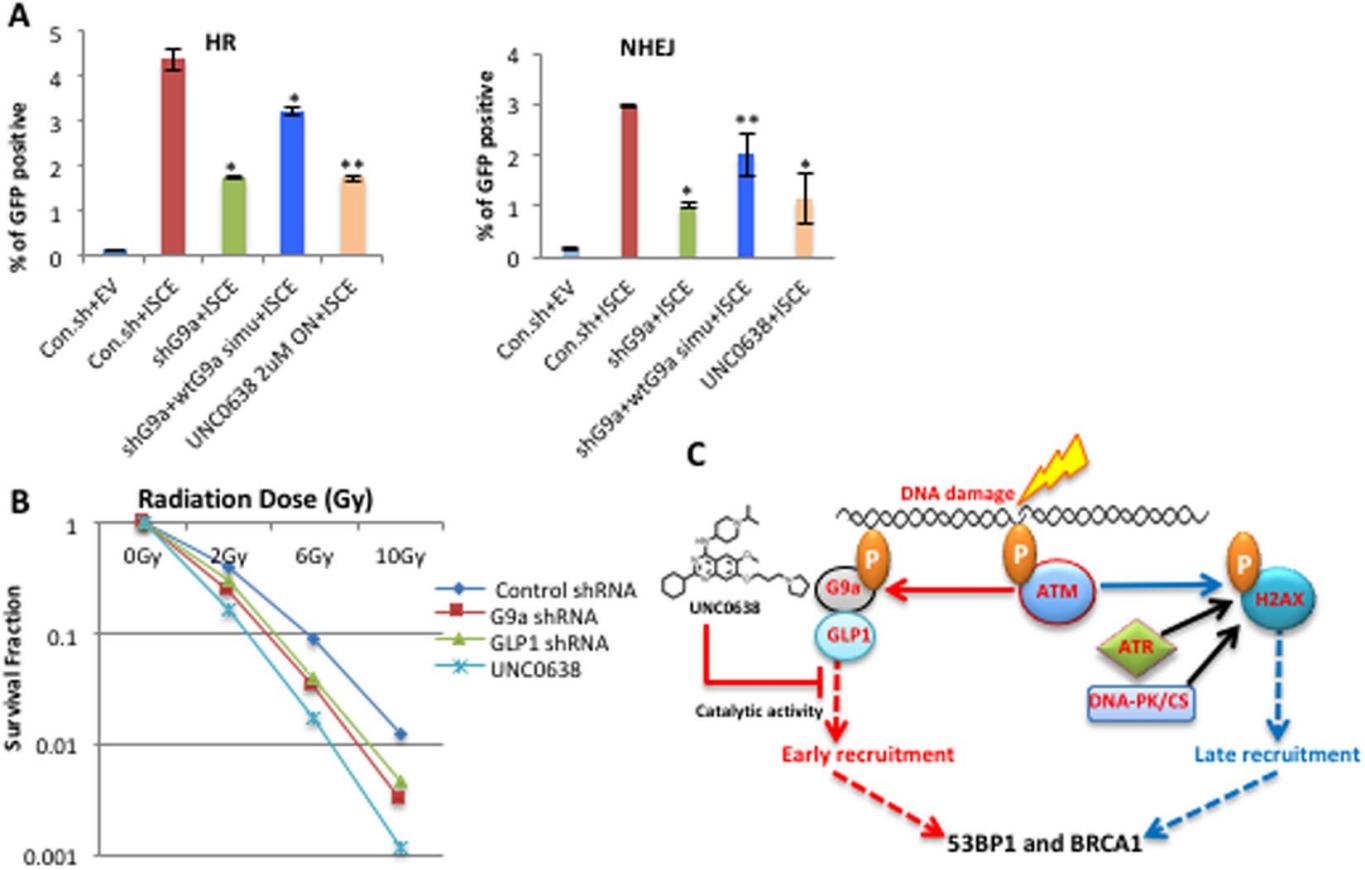
G9a promotes HR and non-homologous end-joining (NHEJ) repair. (**A**) Effects of G9a depletion with shRNA or catalytic inhibition of G9a with UNC0638 on GFP-based reporter assays of HR and NHEJ function are plotted. (**B**) Effects of G9a depletion or inhibition on cellular sensitivity to IR, as analyzed by colony formation in U20S cells. (**C**) A model for the relative roles of H2AX and G9a in early and late recruitment of 53BP1 and BRCA1. P, Phosphorylation. Data are expressed as the mean ± SEM of at least three experiments. **P < 0.01, *P < 0.05. Scale bar 5 μm.

To explore the functional role of G9a in DNA double strand break repair, experiments were performed with U2OS-DR/U2OS280 cell lines containing integrated tandem GFP-based reporter of HR^33^ or NHEJ^34^ activity. Stable knockdown of G9a using shRNA led to a decrease in the efficacy of both HR and NHEJ-mediated repair (Fig. 7A). The reduction in HR or NHEJ caused by G9a loss could be partly rescued by ectopically expressed wt G9a (Fig. 7A). Catalytic inhibition of G9a with UNC0638 treatment also led to a decrease in both HR and NHEJ similar to that seen with G9a knockdown (Fig. 7A). Of note treatment with UNC0638 has been shown not to affect cell cycle progression^19^. Stable knockdown of G9a or GLP1 by shRNA in U2OS cells or treatment with UNC0638 both led to decreased survival after treatment with IR, as measured by clonogenic assay (Fig. 7 panel B). This finding demonstrates that the loss of G9a leads to increased sensitivity to IR-induced DNA breaks.

## Discussion

The G9a protein methyl transferase has been shown to have multiple roles in epigenetic regulation and development. Some studies have suggested a role for G9a in DNA repair pathways, including the observation that G9a is a substrate for ATM/ATR^20^. Inhibition of G9a with UNC0638 has been shown to induce radio-sensitivity and impair NHEJ^19^ induce aneuploidy^35^ and activation of CHK1 and CHK2^36^ and may disrupt BRCA1 recruitment to IRIF. However, the mechanisms underlying these findings are not clear. We now have demonstrated a direct role of G9a in the DNA repair process.

We have shown that G9a is rapidly recruited to sites of DNA damage in an ATM-dependent fashion. Specifically, phosphorylation of G9a at serine 569 by ATM is required for this recruitment. Other regions of G9a may also contribute to efficient localization to DNA breaks, as loss of the SET and Ankyrin repeat domains did reduce, but not eliminate, recruitment. Intriguingly G9a recruitment is also intact in H2AX −/− cells and MDC1 −/− cells, suggesting that rapid recruitment of phospho-G9a to DNA breaks is independent of these factors.

G9a has multiple potential histone substrates including H3K9, H3K27, and H3K56, as well as non-histone protein substrates. Chromatin immunoprecipitation experiments demonstrate that induction of a DNA double strand break leads to increased H3K9me2 and H3K56me1 in neighboring chromatin (Fig. 5, panel D). Catalytic inhibition of G9a significantly reduces these modifications at these sites (Fig. 5, panel D). This suggests that G9a recruitment to DNA breaks may induce local histone methylation at H3K9 and H3K56. However, G9a may also methylate other non-histone proteins in this setting. DNA breaks have been shown to induce local transcriptional silencing in an ATM-dependent manner^37^. Further investigation is required to determine if G9a activation and H2K9me2 is involved in this process.

We have shown that loss of G9a does not affect induction of H2AX phosphorylation or recruitment of MDC1 to DNA breaks suggesting it functions downstream of these proteins. Loss of G9a or catalytic inhibition of G9a activity does abolish the early recruitment of RNF168, 53BP1 and BRCA1 to induced DNA breaks. As RNF168 ubiquitylation activity is required for recruitment of both 53BP1 and BRCA1, our findings suggest that G9a may function upstream of RNF168 in the DNA damage signaling.

Protein methylation and demethylation has been shown to play an important role in DNA repair^38^. For instance, JMJD1C demethylates K45 of MDC1 and facilitates RNF8-dependent polyubiquitination and RAP80-BRCA1 recruitment^39^. Likewise, another demethylase LSD1, which has been shown to regulate H3K4 demethylation, is also recruited to sites of DNA breaks, where it interacts with RNF168 and is required for efficient recruitment of 53BP1 and BRCA1^40^. The functional interaction between these enzymes and G9a in the repair process is not clear, although G9a is required for RNF168 recruitment at early time points. Further work is required on identifying key histone and non-histone substrates of these enzymes in the setting of DNA repair and their functional role in the repair process. However, these findings suggest that protein methylation, is dynamically regulated during the DNA repair process.

In H2AX−/− MEFS early recruitment of 53BP1 and BRCA1 to laser scissors induce micro-irradiation and to IRIF is intact, although later accumulation of these proteins is abolished^31,32^. The mechanism underlying this early H2AX-independent recruitment of DNA repair proteins is not established. Our data suggest that G9a activity is required for this early H2AX-independent recruitment of both 53BP1 and BRCA1 to DNA breaks. In other words, G9a activity is critical for early recruitment of 53BP1 and BRCA1 to DNA breaks, while H2AX phosphorylation is critical for their late recruitment (see Fig. 7C).

It has been reported that UNC0638 treatment disrupts BRCA1 and BARD1, but not 53BP1, recruitment to IRIF; this was based on observation at 1 hr^41,42^. Our time course analysis demonstrates that UNC0638 treatment does abolish 53BP1 IRIF seen at earlier time points (30 min), but recovers by 1 hr, while BRCA1 IRIF remain abolished at 1 hour but recover by 4 hours. This observation highlights the importance of performing time course analysis of recruitment of DNA repair factors as recruitment may vary significantly over time for different factors and different conditions.

Inhibition of G9a activity leads to impairment of both HR and NHEJ, and induces radio-sensitivity. This confirms prior reports that G9a inhibition impairs DNA NHEJ activity^19^. Small molecule inhibitors of G9a/GLP may be potentially useful agents to target the DNA repair pathway. G9a inhibitors may be particularly useful in the setting of pre-existing DNA repair defects that impair late recruitment of DNA repair factors. Critical roles played by G9a and GLP1 in various diseases have led to the hypothesis that these proteins signify valuable future therapeutic targets. Several small molecule inhibitors have been developed to exclusively block the activity of these enzymes, representing promising therapeutic tools in the treatment of human malignancies^43,44^.

## Materials and Methods

### Cell culture

HeLa, U2OS and HEK293Tcells were obtained from the American Type Culture Collection (ATCC; Rockville, MD) were cultured in DMEM (Invitrogen) supplemented with 10% FBS (HyClone) as recommended. H2AX^+/+^ and H2AX^−/−^ MEFs were a gift from Andre Nussenzweig, and MDC1 proficient and deficient MEFs were a gift from Zhenkun Lou. GM00200 (ATM + / + ) and GM09607 (ATM −/−_) were obtained from Coriel. Cells were grown at 37 °C and in a humidified chamber with 5% CO_2._

Drugs and Antibodies: G9a inhibitor UNC0638 (Sigma-Aldrich), ATM kinase inhibitor KU-55933 (Astra-Zeneca/Kudos Pharmaceuticals (Cambridge, United Kingdom), 0.5 μM), Neocarzinostatin (NCS, Sigma Aldrich, 200 ng/ml,), and ATR inhibitor VE-821 (Cayman chemicals, 3 μM). Antibodies used were: G9a (Cell Signaling Technology), GLP1 (Bethyl Laboratories), phosphorylated (Ser139) H2AX (EMD Millipore), 53BP1 (Bethyl Laboratories), BRCA1 (Gift from Bing Xa), BARD1 (Santa Cruz Biotechnology), anti-PAR (Travigen), SUMO (Santa Cruz Biotechnology), H3K9me2 (EMD millipore), Flag (Sigma Aldrich), SPOC1 (Sigma Aldrich), K63-ubiquitin (EMD Millipore), GFP (Abcam) and GAPDH (Abcam), For immunofluorescence experiments, the secondary antibodies used were: anti-mouse (115-035-068, 1:20,000) and anti-rabbit (711-035-152, 1:20,000) from Jackson ImmunoResearch Labs.

### Plasmids construction of full-length human G9a plasmid encoding enhanced GFP and point mutations

Flag tagged full length Human G9a and deletion constructs are gift from Eiji Hara, EGFP-G9a, phospho-mutant EGFP-G9a S569A and G9a silent-mutant were generated as bellow. The pcDNA3 Flag tagged full-length human G9a cDNA was modified by insertion of an eGFP cassette into the KpnI site using following primers: KpnI5GFP 5′-GAG GTA CCA TGG TGA GCA AGG GCG AG-3′ and KpnI3GFP 5′-GAG GTA CCC TTG TAC AGC TCG TCC-3′. The phosphorylation mutation (S569A) was generated using QuikChange site-directed mutagenesis kit (Agilent Technologies) using these primers (forward primer 5′-ATT CTG CAG TCG ACG GTA CCA TGG CCG CCG CCG AT-3′ and reverse primer 5′-ATT CTG CAG TCG ACG GTA CCA TGG CTG CCG ATG AAG GC-3′). Similar methods were used to generate shRNA-resistant silent mutations in full-length flag-tagged human G9a cDNA using following primers: Sense: 5′-cagaggagccaccgaaagggttcatgggtctttggggga-3′ and antisense: 5′-tcccccaaagacccatgaaccctttcggtggctcctctg-3′. All constructs were sequence verified. GFP-RNF8 and RNF168 constructs are gift from Jiri Lukas, and shRNA constructs for G9a (TRCN0000036054) and shGLP1 (TRCN0000115670) were purchased from Sigma Aldrich.

Generation of G9a phospospecific antibody: Rabbit polyclonal antibodies that recognize the S569-phosphorylated G9a were generated by Genscript (Piscataway, NJ) against keyhole limpet hemocyanin (KLH)- conjugated G9a phospho-peptide (CTAAPAPPPL{pSER}QDVP) and double affinity purified.

Laser microirradiation and IRIF: Cells were grown in Lab-Tek chamber slides (Nalge®Nunc™ International, Naperville, IL) and sensitized with 10 μM 5-iodo-2-deoxyuridine (Sigma, St. Louis, MO) for 24 hr before irradiation. Laser microirradiation was performed using a PALM (Photo-activated Localization Microscopy, Bernried, Germany) UV-A pulsed solid-state laser (100 Hz, l 1⁄4 355 nm; P.A.L.M. Microlaser) integrated to a Zeiss Axiovert 200 microscope (Carl Zeiss AG) on a custom-designed granite plate. A LabTek chamber slide with live-cells was mounted on the microscope stage integrated with the PALM Microlaser workstation. The cells were visualized under visible light, laser-targeted nuclei were selected using the Zeiss software (PALM Robo V4.6), and the nuclei were subsequently irradiated with a pulsed solid-state UV-A laser coupled to the bright-field path of the microscope focused through an LD 40× or 60× objective lens to yield a spot size of ∼1 μm. The laser output was set to 50%, which was the lowest power that reproducibly gave a focused pH2AX/53BP1-positive nuclear stripe. Typically, an average of 100 cells were micro-irradiated within 2 to 5 min, and each cell/nucleus was exposed to the laser beam for less than 500 ms. Striped cells were immediately returned to a 37 °C and 5% CO_2_ incubator and fixed at different time points. Ionizing radiation was performed on Cells seeded in triplicate chamber slides and irradiated with a ^137^Cs γ-ray source (Best® Theratronics Gamma Cell 40-Exactor) with a dose rate of 1.08 Gy min^–1^ at room temperature. Cells were treated with mean irradiation doses from 0 to 10 Gy following incubation and fixation. Quantitation was performed as described previously^24^.

### Immunofluorescence staining of cells

Cells were washed twice with PBS fixed in 4% paraformaldehyde and processed for IF as described previously^24^.

### TALEN-driven DNA Double-strand breaks

Transcription activator-like effector nucleases (TALENs) target pair were purchased from Addgene plasmid repository (Plasmid #35431 and Plasmid #35432) and used as described elsewhere^23^. The left and right TALEN plasmids were transfected (FuGene®6; Roche) in to U2OS cells and incubated for 24 hr, followed by the ChIP protocol as explained bellow.

### Chromatin immunoprecipitation (ChIP)

ChIP assays were carried out according to the protocol described previously^24,45^. The average fragment size of soluble chromatin fragments after sonication was ∼500 bp. *In vivo* cross-linking, chromatin purification and immunoprecipitations were as described previously^46^. A total of 150 µg of chromatin was immunoprecipitated using 2 µg of anti-γH2AX or without antibody (mock). The other antibodies used were obtained from various commercial suppliers mentioned in antibodies section. For ChIP-qPCR, immunoprecipitated and input DNA were analyzed in triplicate by real time quantitative-PCR (primer sequences as mentioned previously^24^). Real-time PCR was performed using Applied Biosystems® SYBR® Green PCR Master Mix on a Stratagene® MX3005 P instrument, and quantitative reverse transcription-PCR (RT-PCR) data were analyzed by using the 2ΔΔ^−CT^ method as described previously^47^.

### shRNA knockdowns

Sigma MISSION shRNA targeting and non-targeting control plasmids were used according to manufacturer’s instructions. Lentiviral particles were prepared using cationic lipid-mediated transfection (Invitrogen^TM^ Lipofectamine® 2000) of sub-confluent HEK293T cells cultured in DMEM plus 10% FBS. For a 10-cm dish, lentiviral vector (10 μg) was co-transfected with the lentiviral packaging vector (psPAX2, Addgene, 12260) and the amphotropic envelope (pMD2.G, Addgene, 12259). 24 hr post transfection media containing viral-particles were filtered (Millipore Steriflip-GP Filter, 0.45 um Durapore PVDF) and tittered (Takara Clontech Lenti- X^TM^GoStix^TM^). Transduction of viral particles conducted by adding viral particles to the target cells containing 8 μg/mL polybrene and spinoculation^48,49^ was performed by centrifugation for 2 hr at 1000 g at 4 °C and left the plates at 37 °C, 5% CO2 incubator. As the viral particles contain puromycin-resistance gene, determination of functional viral titer was performed by drug-resistance colony assay as described^50^. Three days post-transduction, cells were pelleted and re-suspended in fresh complete medium containing 2 μg/mL puromycin. Along with the transduced cultures, a non-transduced culture was also selected in puromycin-containing medium to serve as a control for judging when the transduced cells emerged from selection.

### RNA extraction, reverse transcription and PCR

Total RNAs were extracted from the cell lines using TRIzol® reagent (Life Technologies, Grand Island, NY, USA). The extracted RNA (2 μg) was reverse transcribed using random primers (Promega®, Madison, WI, USA) and M-MLV reverse transcriptase (Promega®) for 60 min at 37 °C, the reaction was inactivated for 15 min at 70 °C, and the product was stored at −20 °C until use. The PCR primers were listed in the table below. The PCR cycling conditions were as follows: 95 °C for 2 min, followed by 27 cycles of 95 °C for 30 s, 53 °C for 30 s and 72 °C for 8 s, then extension at 72 °C for 5 min ABI 9800 Thermal Cycler (Applied Biosystems®, Carlsbad, CA, USA). PCR products were checked by 1.5% agarose gel electrophoresis to examine the mRNA expression level of G9a, GLP1 and GAPDH. PCR reactions containing either no template or no reverse transcriptase were used as negative controls and GAPDH was used as the positive control. The primer sequences 5′ to 3′ were following: G9a Forward CCA AGA GAA AGG CTG AAG G and G9a Reverse ACT TGG CAT CTC CAG CAC; GLP1 Forward TTC AAG CAA TTT TCC TGT CT and GLP1 Reverse CAT TAA TCC CAG CAC TTT GG; GAPDH Forward ATC ACC ATC TTC CAG GAG C and GAPDH Reverse TAG GAA CAC GGA AGG CCA TG.

*In-Situ* Proximity Ligation Assay: *In-situ* proximity ligation assay (PLA®, Olink Bioscience®, Uppsala) in combination with immunofluorescence microscopy was performed as described previously^51^ to detect protein–protein interactions

### Western blotting

Cells were lysed in RIPA buffer (50 mM TrisHCl pH 8, 150 mM NaCl, 1% NP40, 0.5 M Sodium Deoxycholate, 0.1% SDS, 25 mM NaF, 1 mM Na_3_VO_4_, complete protease inhibitor cocktail (Roche®)) for 30 min on ice. Protein concentration was measured using the Bio-Rad® colorimetric protein assay kit based on a modified Bradford and Lowry assay method. Protein lysates (20–40 μg) were denatured in Laemmli loading buffer, resolved by gradient SDS-PAGE (4 to 15% gels) and transferred to polyvinylidene difluoride (PVDF) membranes.

### Co-Immunoprecipitation

Either wildtype G9a or S569A phospho-mutant tagged with GFP was expressed in HeLa cells and treated with NCS or vehicle. Nuclear extracts (Activ Motif) were immunoprecipitated with GFP antibodies and processed for Western blotting.

### Clonogenic survival assay

The procedures have been described in detail elsewhere^24,52^.

### DNA double strand break repair (DBSR) assays

To measure homologous recombination (HR) or non-homologous end joining (NHEJ) efficiency, specific cells were stably selected after either lentiviral particle infections (shG9a or shGLP1) or drug treatment along with specific controls. HR assay were performed as described previously using the U2OS/DR-GFP reporter cell line^33,53^, whereas U2OS EJ5-GFP cells were used for NHEJ assay^54^. Cells were seeded at 2 × 10^5^ cells per well in 6-well plates and transfected the next day with various plasmids mixed with 3.6 μl of Lipofectamine 2000 (Invitrogen) in a 1-ml culture medium without antibiotics. Transfections included 0.8 μg of I-SceI expression vector (pCBASce) and either 0.4 μg or 1 μg of a second plasmid: empty vector (pcDNA3.1), an expression vector for G9a silent-mutant (simut), or an expression vector for wild-type G9a (wtG9a). Four hr after transfection, the medium was changed, and 3 days after transfection, the percentage of GFP-positive cells from each transfection were quantified by using the Cytomics FC 500 Series flow cytometer (Beckman Coulter). The data were analyzed by using CXP software (Beckman Coulter).

### Statistical analysis

All the error bars represent the standard errors of the mean (SEM) from at least three or more independent biological replicates unless otherwise indicated. Comparisons between two groups were done using unpaired Student’s t-test and P < 0.05 was considered statistically significant.

## Electronic supplementary material

Supplemental Information
