# Supplementary material for: Protein-lysine methyltransferases G9a and GLP1 promote responses to DNA damage
Source: Sci Rep. 2017 Nov 30;7:16613. doi: 10.1038/s41598-017-16480-5 (PMC5709370; doi:10.1038/s41598-017-16480-5)
Supplement: Supplementary file 1 — Supplemental Information [file 41598_2017_16480_MOESM1_ESM.pdf]

Protein-lysine methyltransferases G9a and GLP1 promote responses to DNA damage

Vasudeva Ginjala[1], Lizahira Rodriguez-Colon[1], Bratati Ganguly[1], Prawallika Gangidi[2], Paul Gallinia[1], Husam Al-Hraishawi[1], Atul Kulkarni[1], Jeremy Tang[1], Jinesh Gheeya[1], Srilatha Simhadri[1], Ming Yao[1], Bing Xia[1] and Shridar Ganesan[1*]

Department of Medicine, Rutgers Cancer Institute of New Jersey[1], Rutgers University, 195 Little Albany street, New Brunswick, New Jersey 08903, USA.

Cornell University, College of Engineering, Department of Biological Engineering[2], 111 Wing Drive, Ithaca, NY 14853-5701, USA

[*]Corresponding Author: Shridar Ganesan

Tel.: (732) 235-5211

FAX: (732) 235-5331

email: ganesash@cinj.rutgers.edu

Supplemental information

Protein-lysine methyltransferases G9a and GLP1 promote responses to DNA damage

Vasudeva Ginjala, Lizahira Rodriguez-Colon, Bratati Ganguly, Prawallika Gangidi, Paul Gallinia[1], Husam Al-Hraishawi, Atul Kulkarni, Jeremy Tang, Jinesh Gheeya, Srilatha Simhadri, Ming Yao, Bing Xia and Shridar Ganesan

Supplemental Figure 1

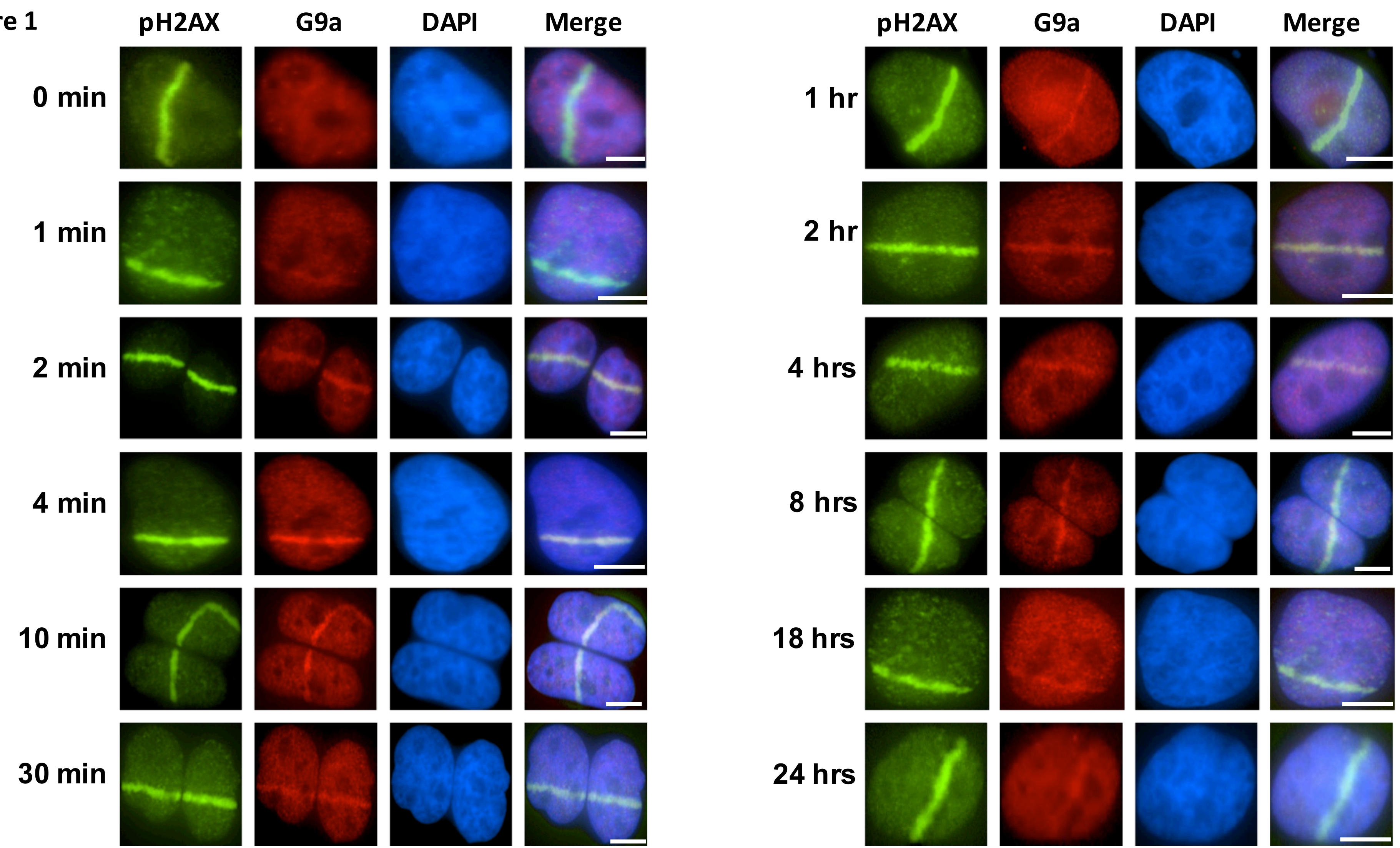

Supplemental information

Protein-lysine methyltransferases G9a and GLP1 promote responses to DNA damage

Vasudeva Ginjala, Lizahira Rodriguez-Colon, Bratati Ganguly, Prawallika Gangidi, Paul Gallinia[1], Husam Al-Hraishawi, Atul Kulkarni, Jeremy Tang, Jinesh Gheeya, Srilatha Simhadri, Ming Yao, Bing Xia and Shridar Ganesan

Supplemental Figure 2

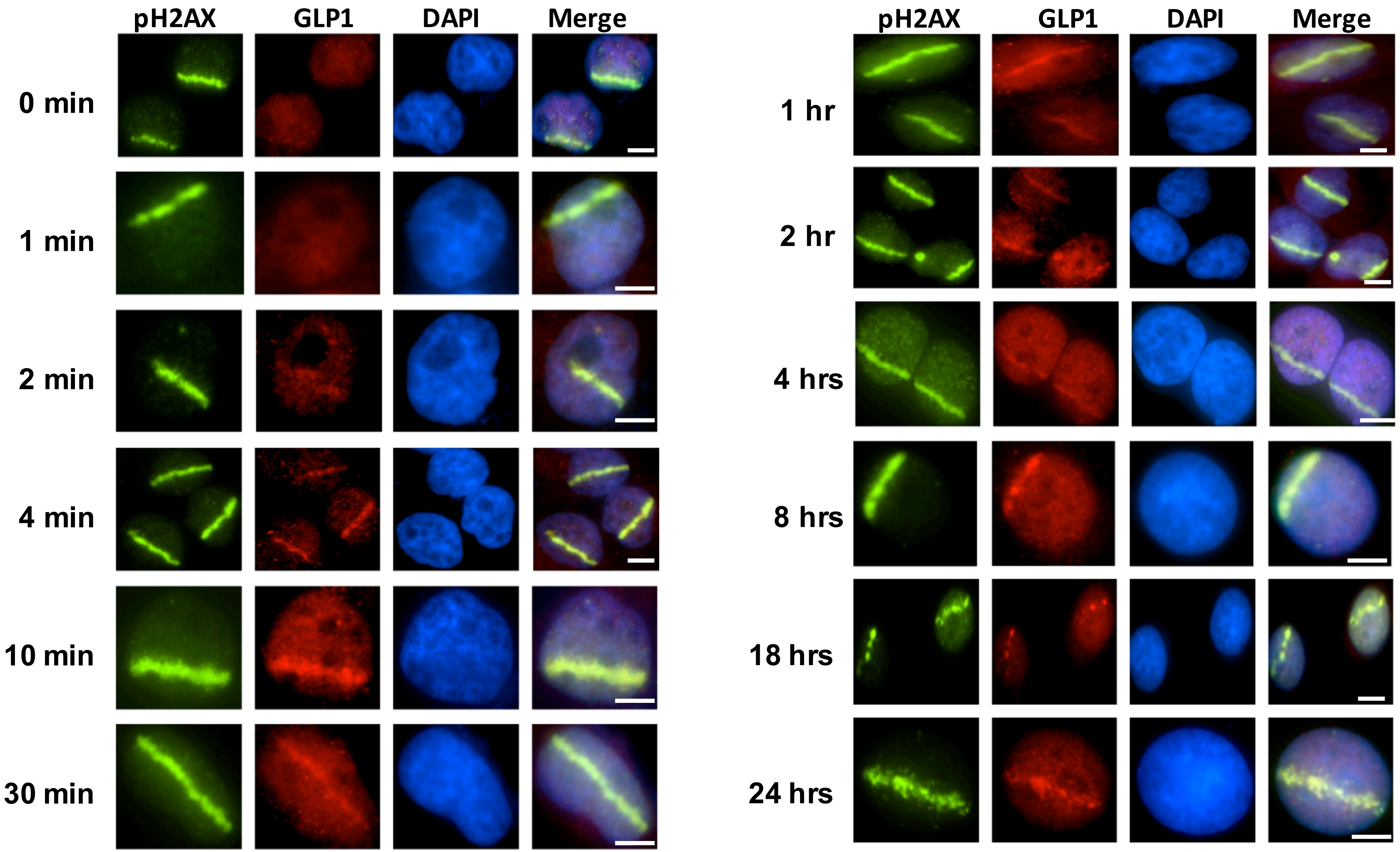

Supplemental information

Protein-lysine methyltransferases G9a and GLP1 promote responses to DNA damage

Vasudeva Ginjala, Lizahira Rodriguez-Colon, Bratati Ganguly, Prawallika Gangidi, Paul Gallinia[1], Husam Al-Hraishawi, Atul Kulkarni, Jeremy Tang, Jinesh Gheeya, Srilatha Simhadri, Ming Yao, Bing Xia and Shridar Ganesan

Supplemental Figure 3

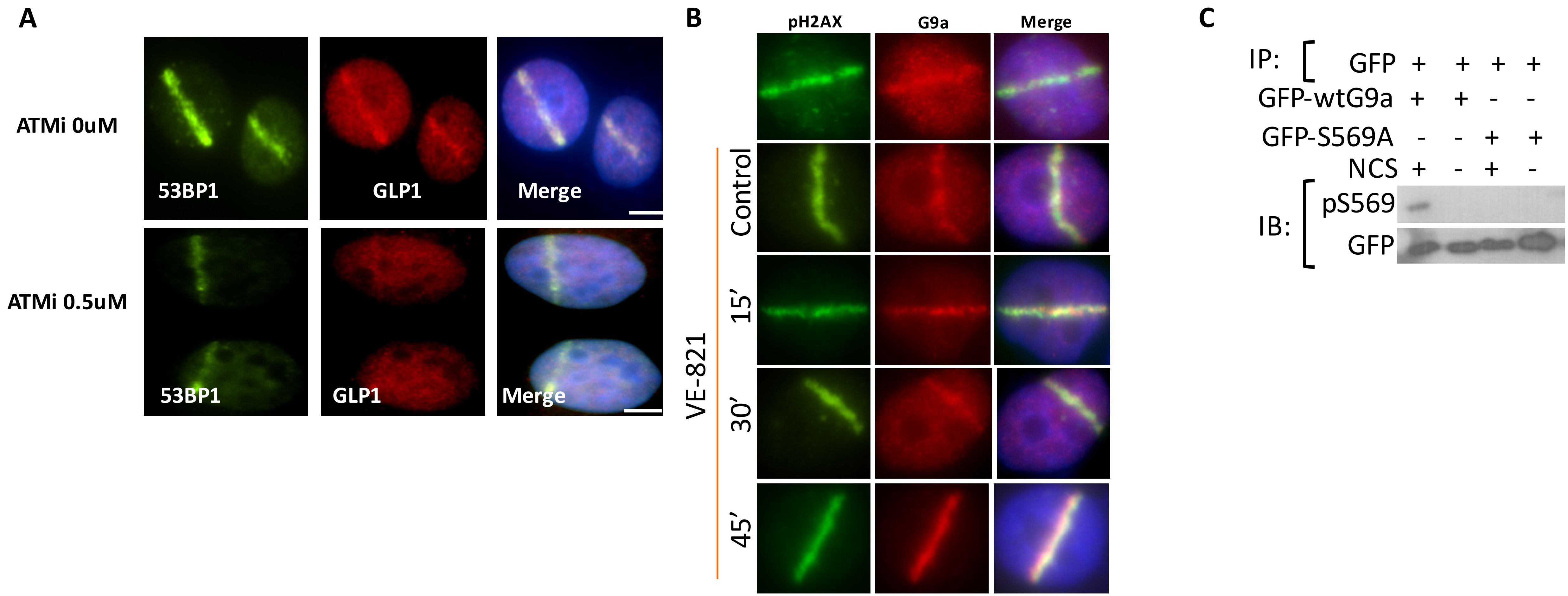

Supplemental information

Protein-lysine methyltransferases G9a and GLP1 promote responses to DNA damage

Vasudeva Ginjala, Lizahira Rodriguez-Colon, Bratati Ganguly, Prawallika Gangidi, Paul Gallinia[1], Husam Al-Hraishawi, Atul Kulkarni, Jeremy Tang, Jinesh Gheeya, Srilatha Simhadri, Ming Yao, Bing Xia and Shridar Ganesan

Supplemental Figure 4

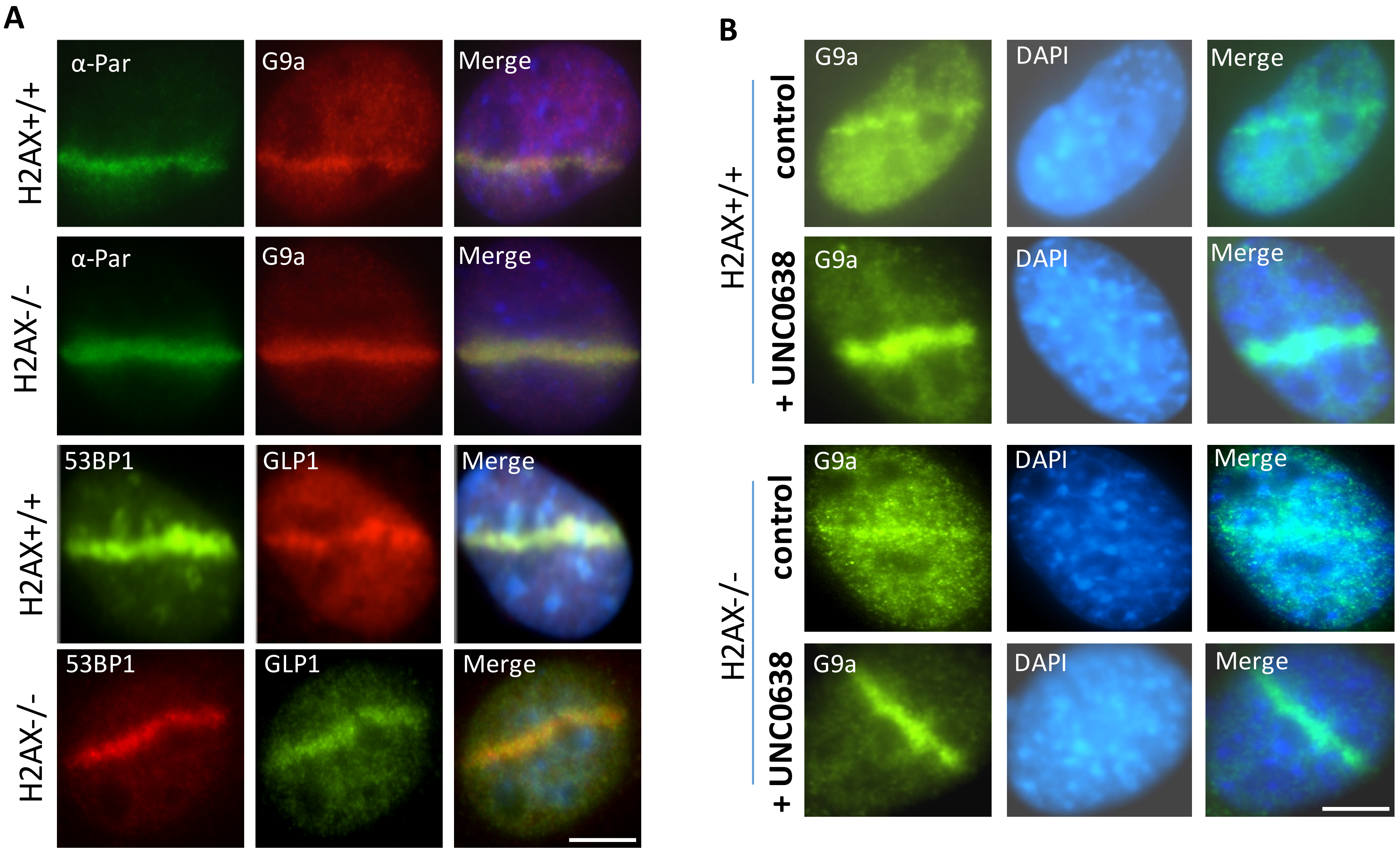

Supplemental information

Protein-lysine methyltransferases G9a and GLP1 promote responses to DNA damage

Vasudeva Ginjala, Lizahira Rodriguez-Colon, Bratati Ganguly, Prawallika Gangidi, Paul Gallinia[1], Husam Al-Hraishawi, Atul Kulkarni, Jeremy Tang, Jinesh Gheeya, Srilatha Simhadri, Ming Yao, Bing Xia and Shridar Ganesan

Supplemental Figure 5

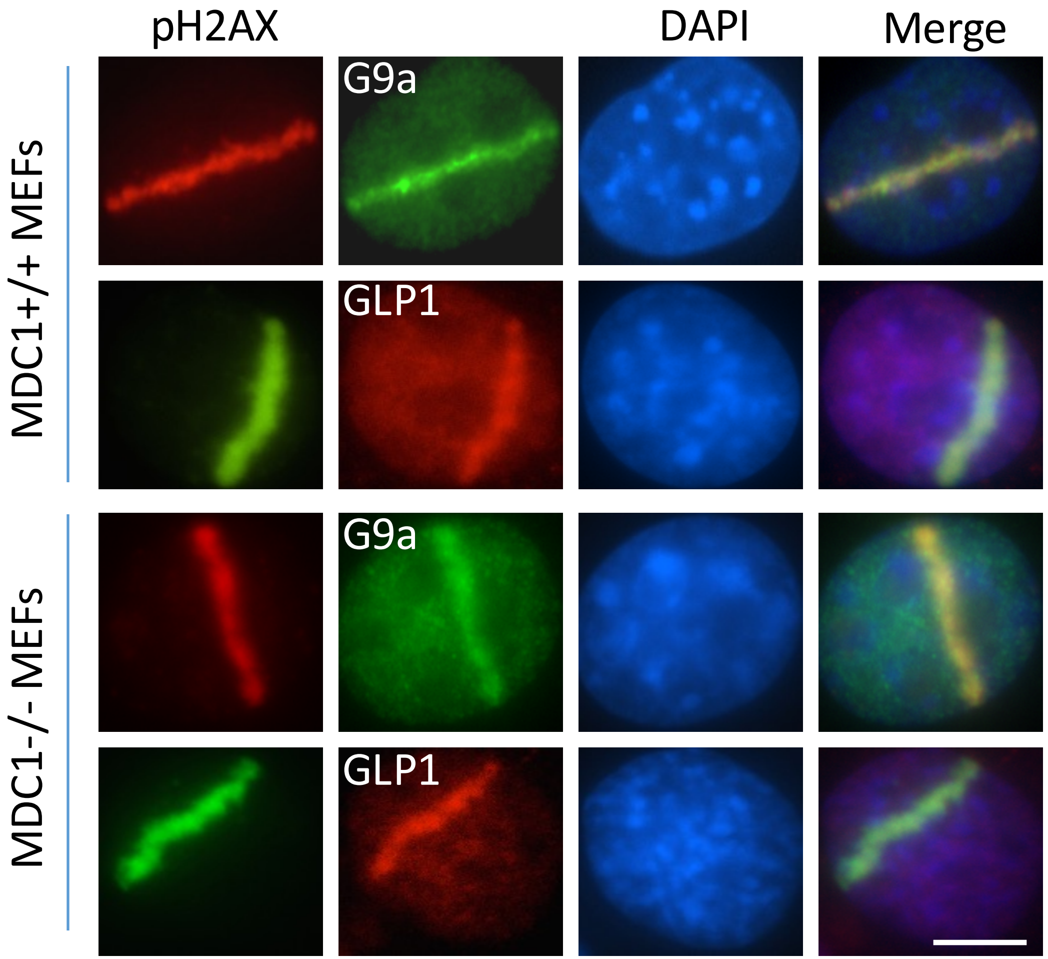

Supplemental information

Protein-lysine methyltransferases G9a and GLP1 promote responses to DNA damage

Vasudeva Ginjala, Lizahira Rodriguez-Colon, Bratati Ganguly, Prawallika Gangidi, Paul Gallinia[1], Husam Al-Hraishawi, Atul Kulkarni, Jeremy Tang, Jinesh Gheeya, Srilatha Simhadri, Ming Yao, Bing Xia and Shridar Ganesan

Supplemental Figure 6

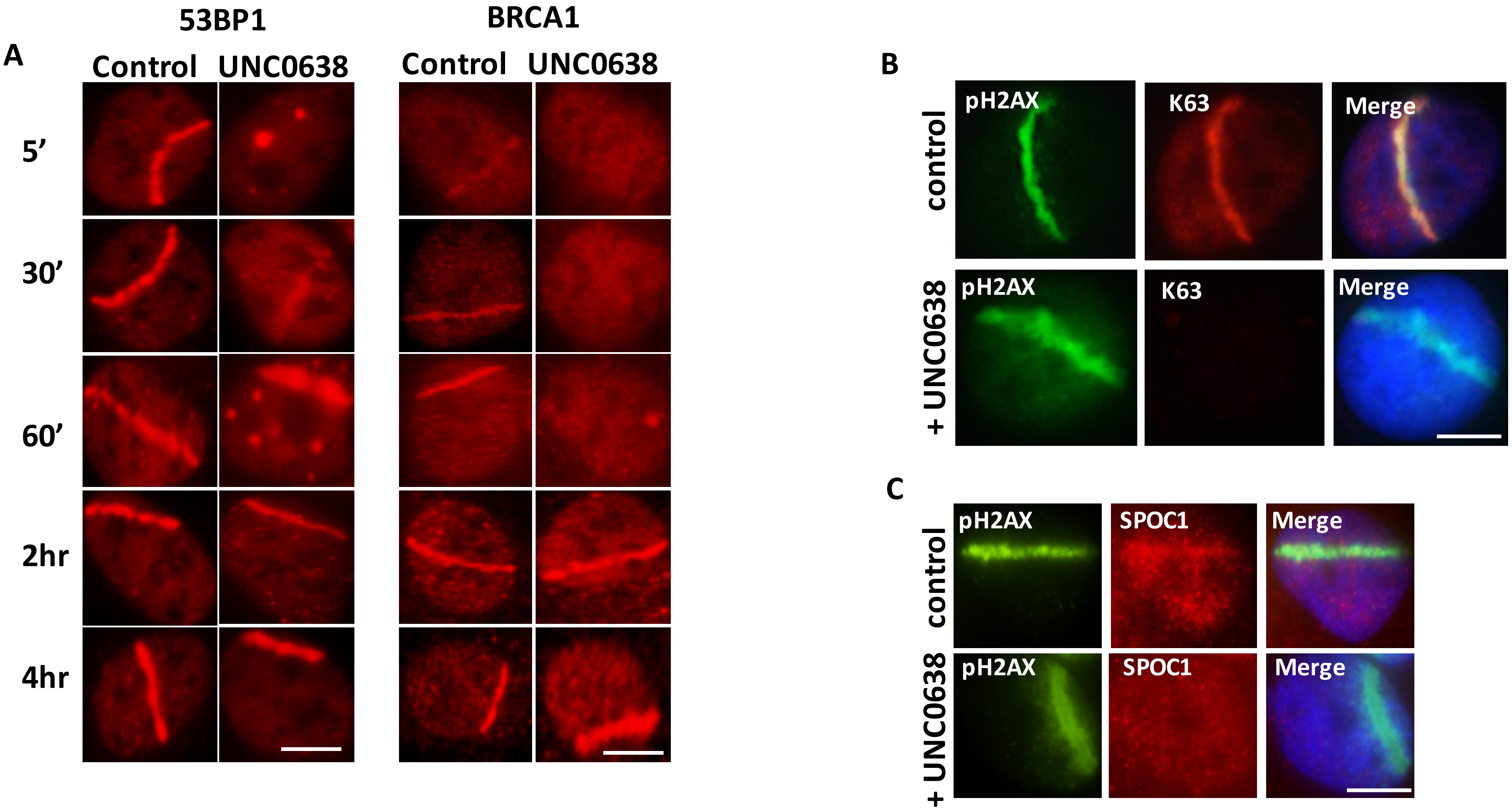

Supplemental information

Protein-lysine methyltransferases G9a and GLP1 promote responses to DNA damage

Vasudeva Ginjala, Lizahira Rodriguez-Colon, Bratati Ganguly, Prawallika Gangidi, Paul Gallinia[1], Husam Al-Hraishawi, Atul Kulkarni, Jeremy Tang, Jinesh Gheeya, Srilatha Simhadri, Ming Yao, Bing Xia and Shridar Ganesan

Supplemental Figure 7

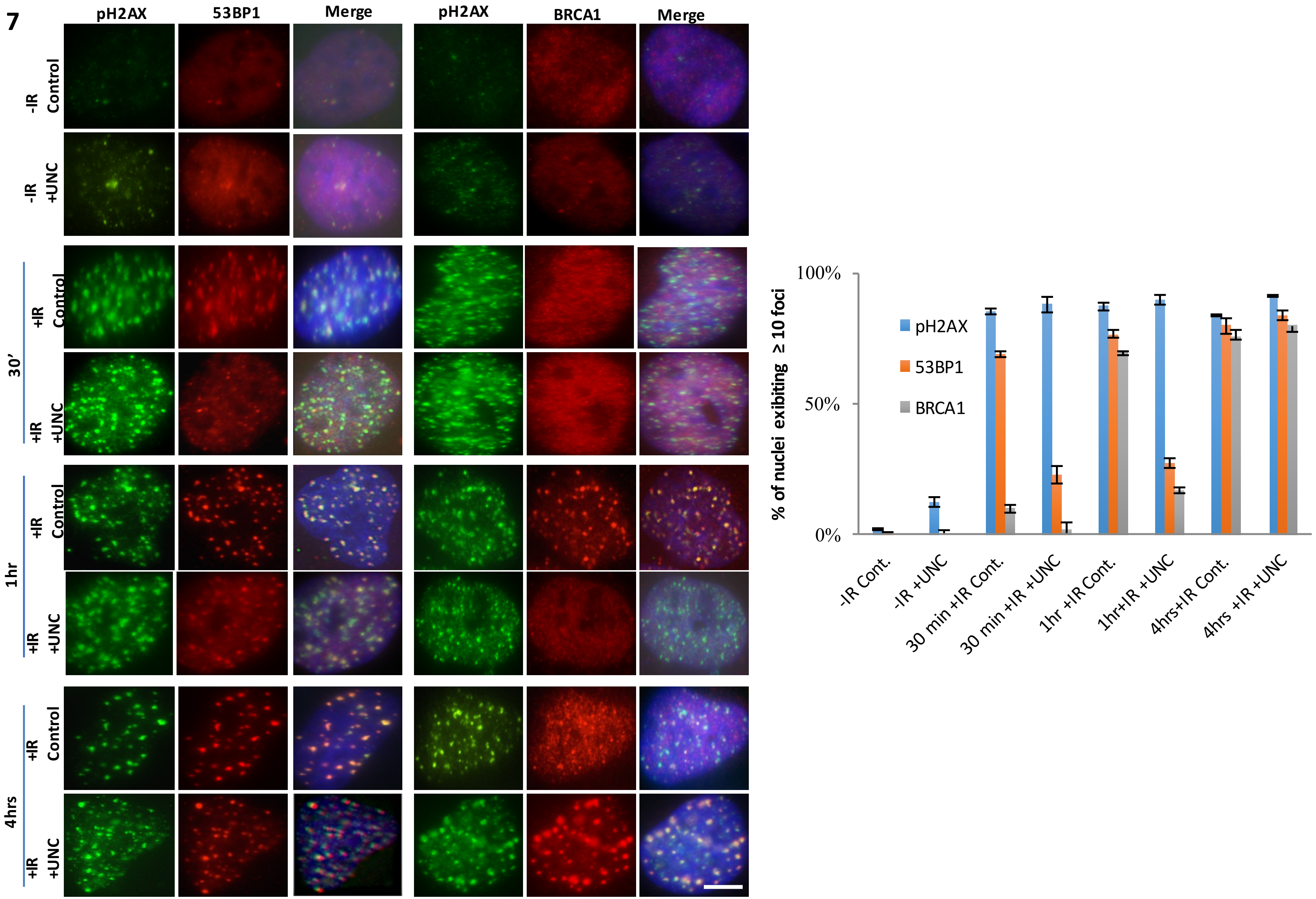

Supplemental information

Protein-lysine methyltransferases G9a and GLP1 promote responses to DNA damage

Vasudeva Ginjala, Lizahira Rodriguez-Colon, Bratati Ganguly, Prawallika Gangidi, Paul Gallinia[1], Husam Al-Hraishawi, Atul Kulkarni, Jeremy Tang, Jinesh Gheeya, Srilatha Simhadri, Ming Yao, Bing Xia and Shridar Ganesan

Supplemental Figure 8

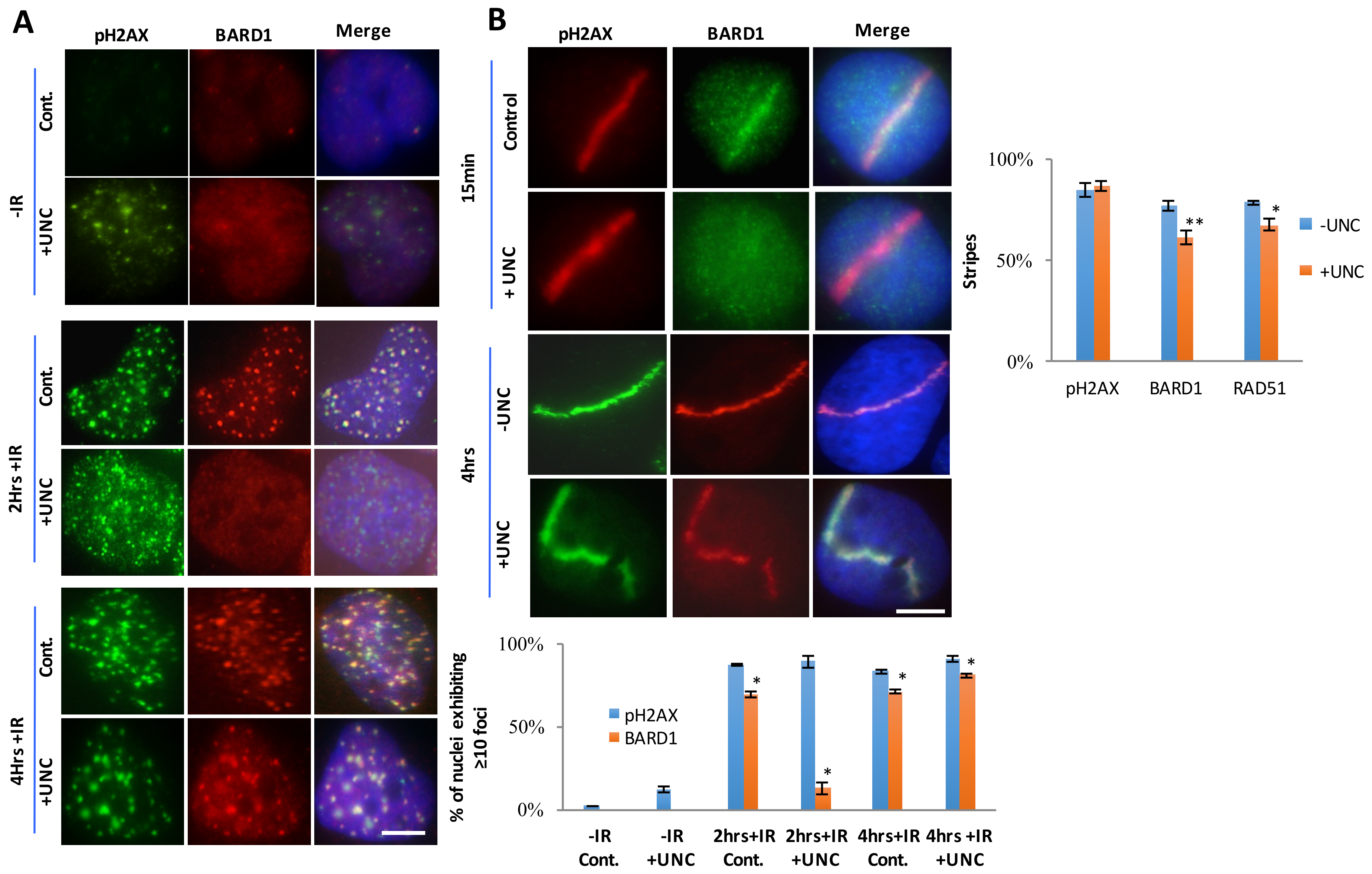

Supplemental information

Protein-lysine methyltransferases G9a and GLP1 promote responses to DNA damage

Vasudeva Ginjala, Lizahira Rodriguez-Colon, Bratati Ganguly, Prawallika Gangidi, Paul Gallinia[1], Husam Al-Hraishawi, Atul Kulkarni, Jeremy Tang, Jinesh Gheeya, Srilatha Simhadri, Ming Yao, Bing Xia and Shridar Ganesan

Supplemental Figure 9

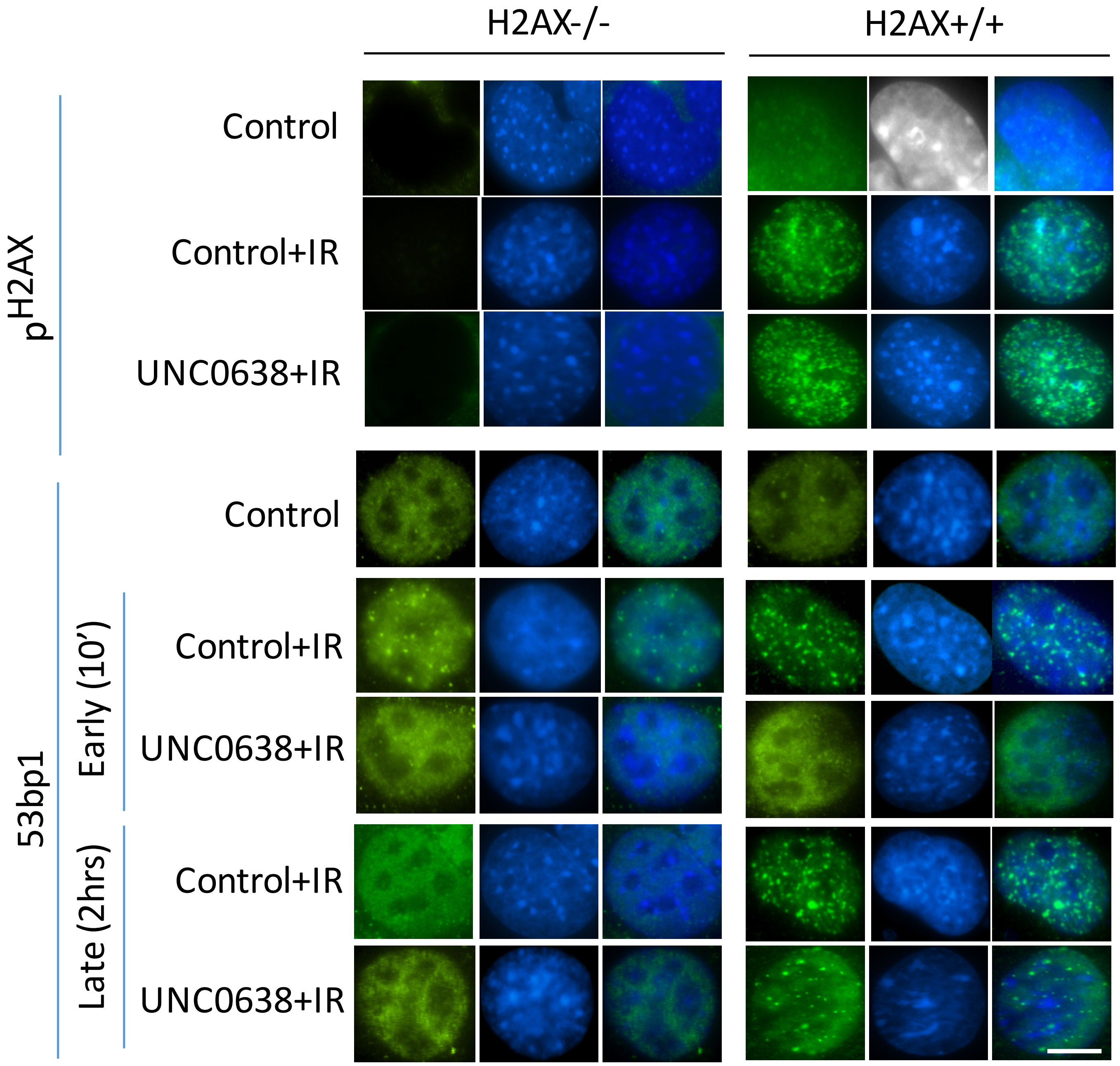

Supplemental information

Protein-lysine methyltransferases G9a and GLP1 promote responses to DNA damage

Vasudeva Ginjala, Lizahira Rodriguez-Colon, Bratati Ganguly, Prawallika Gangidi, Paul Gallinia[1], Husam Al-Hraishawi, Atul Kulkarni, Jeremy Tang, Jinesh Gheeya, Srilatha Simhadri, Ming Yao, Bing Xia and Shridar Ganesan

Supplemental Figure 10

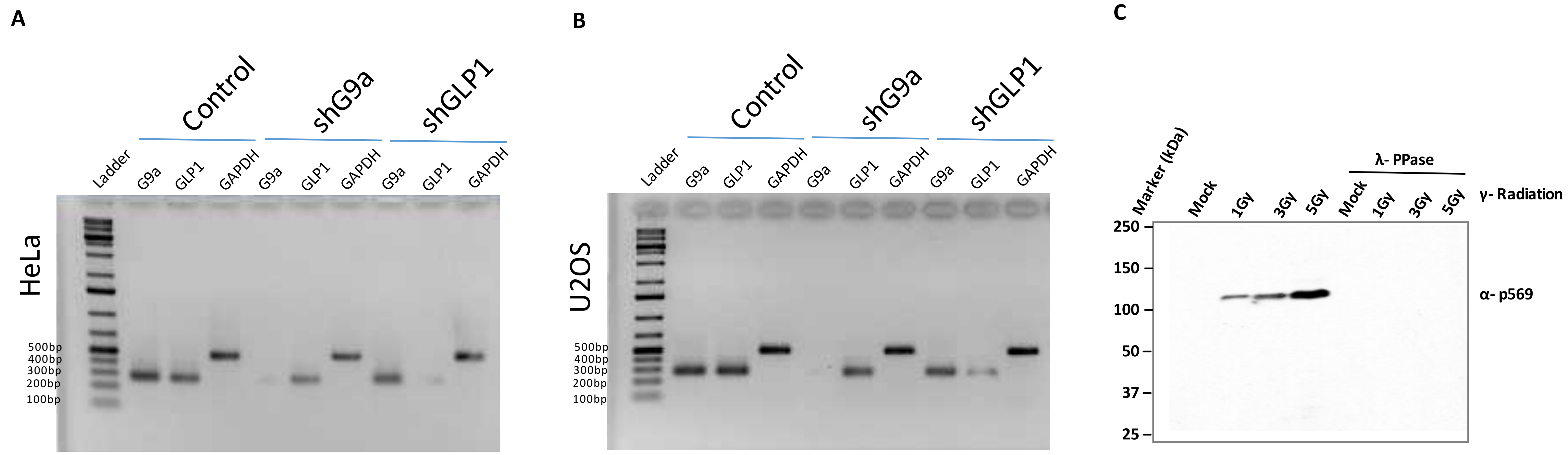

Supplemental information

Protein-lysine methyltransferases G9a and GLP1 promote responses to DNA damage

Vasudeva Ginjala, Lizahira Rodriguez-Colon, Bratati Ganguly, Prawallika Gangidi, Paul Gallinia[1], Husam Al-Hraishawi, Atul Kulkarni, Jeremy Tang, Jinesh Gheeya, Srilatha Simhadri, Ming Yao, Bing Xia and Shridar Ganesan

Supplemental Figure 10

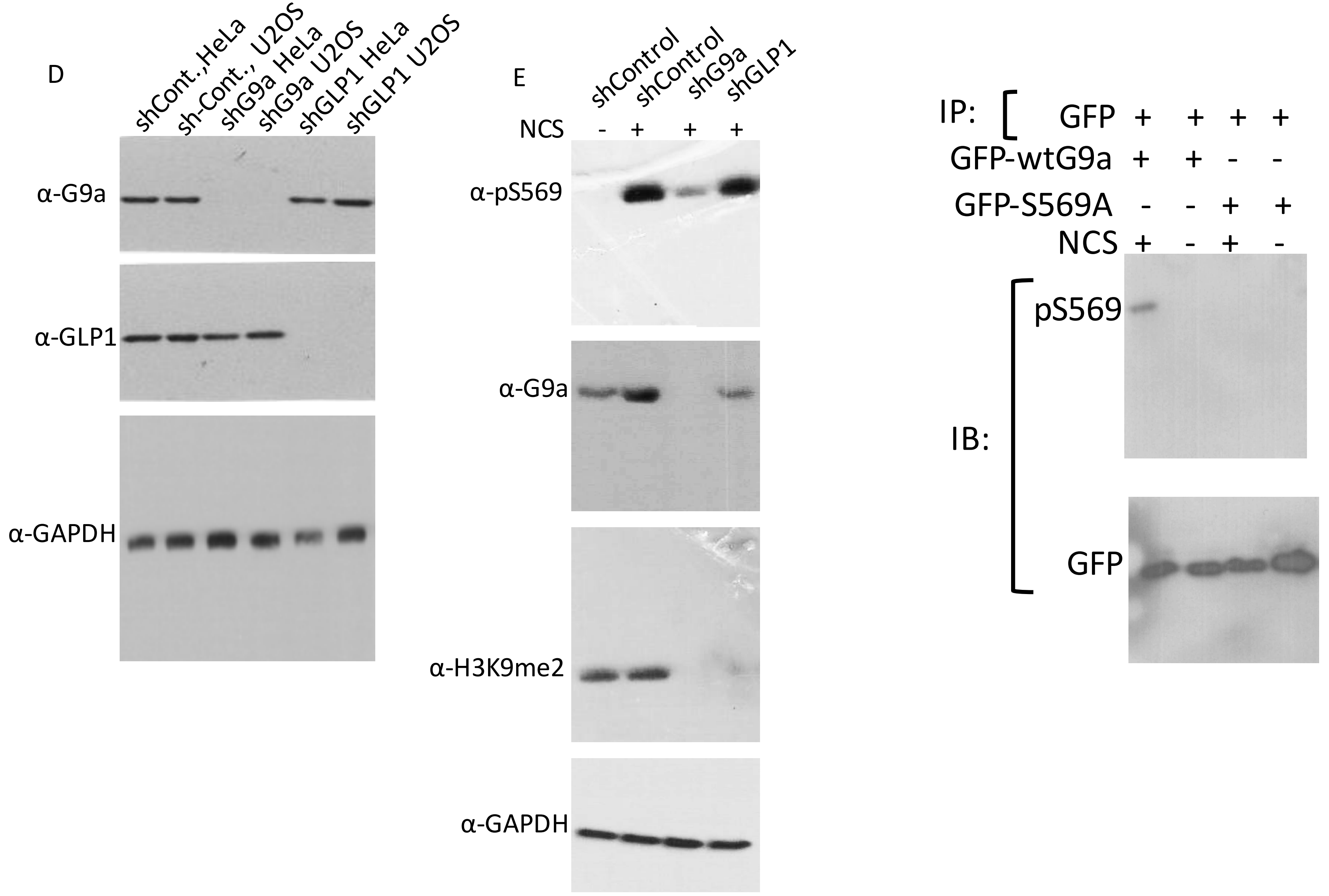

Supplemental Figure Legends

Supplemental Figure. 1. G9a is recruited at the site of DNA damage. An asynchronous population of U2OS cells was micro-irradiated and fixed with paraformaldehyde at different time-points (0-24hrs) and processed for IF using antibodies to γH2AX (green) and G9a (red); DNA was stained with DAPI (blue). Scale bar 5µm.

Supplemental Figure. 2. GLP1 is recruited at the site of DNA damage. An asynchronous population of U2OS cells was micro-irradiated and fixed with paraformaldehyde at different time-points (0-24hrs) and processed for IF using antibodies to γH2AX (green) and GLP1 (red); DNA was stained with DAPI (blue). Scale bar 5µm.

Supplemental Figure. 3. (A) GLP1 localization to DNA breaks is dependent upon ATM. HeLa cells were treated with either 0 u or 0.5 uM ATMi overnight, treated with micro-irradiation, and were fixed in paraformaldehyde and immune-stained for 53BP1 (green), and GLP1 (red). DAPI nuclear staining is shown in blue. Scale bar 5µm. (B) G9a localization to DNA breaks is independent of ATR. HeLa cells were treated with either 0 uM or 3.0uM of the ATR inhibitor VE-821, then treated with micro-irradiation, and after different time points, were fixed in paraformaldehyde and immune-stained for pH2AX (green), and G9a (red). DAPI nuclear staining is shown in blue. Scale bar 5µm. (C) HeLa cells were transfected with constructs expressing either wt-G9a-GFP or S569A-G9a-GFP fusion constructs. Proteins from nuclear extracts of HeLa cells were Immunoprecipitated (IP) with GFP antibodies and processed for Western Blotting with phosho-S569 G9a antibody. Membranes were stripped and re-probed with anti-GFP

antibody for loading control. (D) Western blots of U2OS cells treated with various doses of gamma radiation and probed with indicated antibodies.

Supplemental Figure. 4. (A) G9a or GLP1 recruitment to DNA breaks independent of H2AX. H2AX -/- or H2AX +/+ MEFS were treated with micro-irradiation and after ~10 minutes, fixed and processed for IF using antibodies as shown. (B) G9a localization to DNA breaks independent of H2AX and/or loss of its catalytic activity. H2AX -/- or H2AX +/+ MEFS were treated with micro-irradiation and after ~10 minutes, fixed and processed for IF using anti-G9a antibodies as shown. Scale bar 5µm.

Supplemental Figure. 5. Genetic Loss of MDC1 does not affect G9a or GLP1 localization to DNA Breaks. MDC1 -/- and +/+ MEFs were treated with micro-irradiation and after ~10 minutes, fixed and processed for IF using antibodies to γH2ax and G9a/Glp1. Scale bar 5µm.

Supplemental Figure 6. (A) G9a catalytic activity critical for early recruitment of 53BP1 and BRCA1 to DNA breaks. U2OS cells pre-treated with either 0 uM or 2.5 uM UNC0638 for 2 hours and then micro-irradiated and fixed with paraformaldehyde at different time-points (5 min-4 hrs) and immune-stained for 53BP1 or BRCA1 as shown. (B) G9a Catalytic activity required for proper accumulation of K63 poly-ubiquitination signal at DNA breaks. U2OS cells pre-treated with either 0 uM or 2.5 uM UNC0638 for 2 hours and then micro-irradiated and fixed with paraformaldehyde at 5 min and immune-stained for H2AX and K63 as shown. (C) Catalytic activity of G9a essential for SPOC1 localization to DNA breaks. U2OS cells pre-treated with either 0 uM or 2.5 uM UNC0638

for 2 hours and then micro-irradiated and fixed with paraformaldehyde at 5 min and immune-stained for H2AX and SPOC1antibodies as shown. Scale bar 5μm.

Supplemental Figure 7. Loss of G9a catalytic activity abrogates 53BP1 and BRCA1 early nuclear foci formation. U2OS cells were treated with 0 uM or 2.5 uM UNC0638 for 2 hours, exposed to γ-irradiation (0 or 10 Gy) and fixed with paraformaldehyde at different time-points (30 min-4 hrs). Cells were then immune-stained for γH2AX and either 53BP1 or BRCA1 as shown. Quantification of nuclear foci is shown right side of the images. Each image represents 50 γH2AX-positive cells in three independent experiments and data are expressed as the mean ± SEM. **P < 0.01, *P < 0.05. Scale bar 5μm.

Supplemental Figure 8. G9a catalytic activity is required for early localization of BARD1 to DNA breaks. (A) U2OS cells were exposed to γ-irradiation (0 or 10 Gy) and fixed with paraformaldehyde at different time-points (2hrs-4 hrs) and then fixed and immune-stained for γH2AX and BARD1. (B) U2OS cells were micro-irradiated and fixed with paraformaldehyde at different time-points (15 min-4 hrs) and immune-stained for γH2AX and BARD1. The cells were pre-treated with either 0 uM or 2.5 uM UNC0638 and the quantification of foci and UV-laser strips provided right side of the images. Each image represents 50 γH2AX-positive cells in three independent experiments and data are expressed as the mean ± SEM. **P < 0.01, *P < 0.05. Scale bar 5μm.

Supplemental Figure 9. G9a is required for early, H2AX-independent foci formation of 53BP1. Mouse H2AX+/+ (wild-type) and H2AX-/- (KO) MEFs were irradiated with 10Gy and processed by paraformaldehyde fixation at various times after irradiation and then stained for 53BP1 (green). H2AX -/- cells formed 53BP1 foci at 10 minutes after IR, but

not IRIF are present at later time points. The 53BP1 foci present at 10 minutes after IR treatment in H2AX-/- cells are abolished with UNC0638 2.5 uM treatment. 2.5 uM UNC0638 treated H2AX+/+ cells also failed to form 53BP1 nuclear foci at 10 min after irradiation, but nuclear foci are intact at later time-point (2hrs). Scale bar 5μm.

Supplemental Figure 10. The Full-length gels and blots (A) Full-length agarose gel for the original Fig.2A HeLa. (B) Full-length agarose gel for the original Fig.2A U2OS. (C) Full-length western blot for the original Fig.4B. (D) Full-length western blots for the original Fig. 2B. (E) Full-length western blots for the original Fig.4A.
